# Modulation of TNFα-driven neuroinflammation by Gardenin A: insights from *in vitro*, *in vivo*, and *in silico* studies

**DOI:** 10.3389/fphar.2025.1681403

**Published:** 2025-11-10

**Authors:** Prashsti Chadha, Hiral Aghara, Harshrajsinh Solanki, Manali Patel, Dhrubjyoti Sharma, Vijay Thiruvenkatam, Palash Mandal

**Affiliations:** 1 Department of Biological Sciences, PD Patel Institute of Applied Sciences (PDPIAS), Charotar University of Science and Technology, Changa, Gujarat, India; 2 Department of Biological Sciences and Engineering, Indian Institute of Technology Gandhinagar, Palaj, Gujarat, India

**Keywords:** alcohol-induced neuroinflammation, herbal therapeutics, gardenin A, blood-brainbarrier, TNFα

## Abstract

**Introduction:**

Chronic alcohol consumption is a major contributor to neuroinflammation, oxidative stress, and blood-brain barrier (BBB) disruption, leading to significant neuronal injury. Traditional therapies for alcohol use disorder (AUD) predominantly target behavioral and receptor-based mechanisms, often neglecting the direct pathophysiological impacts of alcohol on brain tissue. This study explores the neuroprotective potential of Gardenin A (GarA), a hexa-methoxylated flavone, in counteracting alcohol-induced inflammation and physiological damage.

**Methods:**

*In vitro* experiments utilized SH-SY5Y neuroblastoma cells treated with varying concentrations of GarA, assessing cell viability, nuclear integrity, oxidative stress, and gene expression. *In vivo* experiments involved the administration of ethanol alongside GarA at doses of 50 and 100 mg/kg body weight to male Wistar rats. Subsequent brain tissue analysis employed histological and immunohistochemical methods to evaluate structural preservation and cellular responses. Key molecular targets were examined, including vimentin, brain-derived neurotrophic factor (BDNF), and Claudin5. Protein levels of inflammatory markers and antioxidant enzymes were quantified using ELISA, providing detailed insights into the biochemical pathways involved. Complementary *in silico* methods, such as molecular docking and network pharmacology, were employed to elucidate the mechanistic interactions and predict potential molecular binding sites.

**Results:**

The treatment with GarA resulted in enhanced neuronal viability and a reduction in ethanol-induced oxidative stress *in vitro*. *In vivo* results demonstrated preservation of brain architecture, attenuation of astroglial reactivity, and significant downregulation of tumor necrosis factor-alpha (TNFα), a key mediator of neuroinflammation. Additionally, GarA was associated with restored BDNF expression and upregulated antioxidant markers like HO-1 and Nrf2, maintaining neurovascular integrity and neurotrophic balance.

**Discussion:**

GarA demonstrates neuroprotective potential, with evidence suggesting modulation of neuroinflammation and oxidative stress that may involve TNFα and BDNF pathways. These promising findings suggest potential therapeutic applications for GarA in addressing alcohol-related neurodegeneration. Future research focusing on clinical trials may prove helpful in validating these preclinical findings. Expanding studies to include diverse animal models and exploring combinatory treatments with existing AUD therapies could enhance understanding and application. Such efforts may pave the way for incorporating GarA into comprehensive pharmacotherapeutic strategies aimed at mitigating the neuropathological effects of chronic alcohol consumption.

## 1 Introduction

Ethanol, a widely consumed psychoactive substance and Class I carcinogen, exerts neurotoxic effects through mechanisms involving oxidative stress, inflammation, and metabolic disruption (World Health Organization, 2024). Chronic exposure to ethanol is a well-established contributor to systemic toxicity and is a major risk factor for neurodegenerative disorders, including Alzheimer’s (AD) and Parkinson’s disease (PD). Globally, reports indicate that alcohol consumption has been steadily increasing over time.

While the liver is the primary site of ethanol metabolism, the brain is both susceptible and metabolically active, with the ability to oxidize ethanol via catalase and cytochrome P450 2E1 (CYP2E1), leading to nearly 80% of the neuronal alcohol metabolism (Beisswenger et al., 1985; Martínez et al., 2001; Galter et al., 2003; Zimatkin et al., 2006; Wilson and Matschinsky, 2020; Wen et al., 2022). Furthermore, astrocytes (a type of glial cell) have acetaldehyde dehydrogenase 2 (ALDH2) which leads to the formation of acetate, which contributes to severe intoxication and alcohol dependence (Carmichael et al., 1991; Volkow et al., 2013; Mews et al., 2019; Jin et al., 2021). Alcohol not only crosses the blood–brain barrier (BBB) but also disrupts its structural integrity, exacerbating neuroinflammatory responses (Vore and Deak, 2022). Hence ethanol’s metabolism in neuronal tissue produces acetaldehyde and reactive oxygen species (ROS), leading to cellular redox imbalance, mitochondrial damage, and membrane destabilization. These mechanisms underlie its classification as a neurotoxic agent in toxicological studies.

The central nervous system (CNS) comprises four main types of glial cells: astrocytes, microglia, oligodendrocytes and their precursor cells, polydendrocytes. All of these are significantly impacted by alcohol exposure in terms of their growth, morphology, physiology, and gene expression (Miguel-Hidalgo, 2018). At low levels, alcohol can transiently activate microglia, eliciting neuroprotective responses. However, chronic exposure results in microglial overactivation and the release of pro-inflammatory cytokines. Among the critical mediators of alcohol-induced neurotoxicity, tumor necrosis factor-alpha (TNFα) plays a central pathogenic role, while proprotein convertase subtilisin/kexin type 9 (PCSK9) has been implicated as a novel neuroinflammatory mediator linking the liver-brain axis. Ethanol exposure upregulates TNFα and PCSK9 expression, which in turn amplifies the inflammatory milieu by stimulating microglial and astrocytic activation (accompanied by increased expression of intermediate filament protein such as vimentin) and downstream cytokines such as monocyte chemoattractant protein-1 (MCP-1), interleukin 1-beta (IL-1β), and interlukin-6 (IL-6) (Ward et al., 2009; Qin and Crews, 2012; Patel and Mandal, 2018; Anand et al., 2023).

Prolonged TNFα signaling contributes to neuronal apoptosis, BBB dysfunction, and synaptic loss - hallmarks of alcohol-induced cognitive decline. Ethanol-induced ROS and RNS production, coupled with ER stress, impairs tight junction proteins and compromises BBB integrity (Varadinova et al., 2019). Studies have shown that alcohol induced neuroinflammation, along with oxidative and endoplasmic reticulum (ER) stress, disrupts tight junctions (TJs) and compromises BBB integrity (Carrino et al., 2021; Wei et al., 2021). This further aggravates the inflammation by allowing the permeation of inflammatory cytokines and other immune cells from the peripheral nervous system (PNS) (Togre et al., 2024). In parallel, ethanol suppresses neurotrophic signaling, particularly the expression of BDNF, impairing neuronal survival, plasticity, and repair mechanisms (Tapia-Arancibia et al., 2001; Raivio et al., 2012; Martín-González et al., 2022).

Researchers worldwide are actively investigating the toxic effects of alcohol on the brain and are exploring potential therapeutic strategies to mitigate its impact. While most studies predominantly target alcohol dependence and withdrawal, fewer have addressed the underlying stress mechanisms in the brain caused by chronic alcohol overconsumption. A limited number of preclinical studies have evaluated natural compounds for their potential to alleviate alcohol-induced oxidative stress and inflammation, including resveratrol (Petrella et al., 2020), olive polyphenols (Carito et al., 2017), saffron and crocin (Bandegi et al., 2014) and minocycline (Motaghinejad et al., 2021). Additional research has focused on preventing or treating alcohol-induced neurodegenerative apoptosis using agents such as nicotinamide (Ieraci and Herrera, 2006) and epigallocatechin-3-gallate (EGCG) (Tiwari et al., 2010). Parallel studies have also explored the neuroprotective effect of these natural compounds in the context of neurodegenerative disorders. For instance, quercetin has been shown to mitigate mitochondrial dysfunction in PD (Kang et al., 2020), while baicalein exerts neuroprotection through its anti-inflammatory, antioxidant and anti-apoptotic properties (Lee et al., 2005; Li et al., 2012).

GarA, (C_21_H_22_O_9_) (molecular weight: 418.4 g/mol) a hexa-methoxylated flavone, is found in various plant sources, including *Gardenia resinifera* Roth. (Toppo et al., 2017). and *Murraya paniculata* (Kinoshita and Firman, 1996). It has previously demonstrated hepatoprotective and anti-hyperlipidaemic properties against metabolism-associated liver disease (MASLD) in *in vitro* and *in vivo* models (Toppo et al., 2017) and against alcohol-induced liver and gut damage in an *in vitro* model (Chadha et al., 2025). GarA has also shown anti-oxidative and neuroprotective properties in a rat model of focal brain ischemia (Zhang et al., 2005), and has been found to facilitate neurite outgrowth in PC-12 cells, suggesting potential therapeutic relevance for neurodegenerative conditions such as AD and PD (Chiu et al., 2013). It has anti-anxiety-like, anti-convulsant, and anti-depressant-like neuropharmacological activities in mice (Alonso-Castro et al., 2020) and anti-neuroinflammatory effects in the *Drosophila* model of PD (Maitra et al., 2021). Most importantly, recently (Hack et al., 2024) uncovered the potential of GarA to cross the BBB and protect against cognitive and motor dysfunction against PD.

Currently, three FDA-approved medications are available for the treatment of AUD, but these drugs primarily function by targeting neural receptors and are associated with notable limitations. For example, some of these drugs are contraindicated in individuals with comorbid conditions—naltrexone and disulfiram in those with advanced liver disease, acamprosate in cases of renal impairment, and disulfiram in patients with cognitive deficits (MacKillop et al., 2022; Agabio et al., 2024). Despite growing interest in flavonoid-based neuroprotection, little is known about their effect on ethanol-induced dysregulation of key molecular targets such as TNFα, PCSK9, MCP-1, Claudins, Vimentin, and BDNF. These markers are closely associated with oxidative stress response, tight junction integrity, and neuroinflammatory signaling. This study investigates the potential of GarA to influence TNFα-driven neuroinflammatory cascades and associated molecular markers in ethanol-induced neurotoxicity, using an integrated *in vitro*, *in vivo*, and *in silico* approach.

## 2 Materials and methods

### 2.1 Materials

Culture media, supplements, antibiotic-antimycotic solution, fetal bovine serum (FBS), 1X phosphate buffered saline (PBS) and rat feed components were procured from HiMedia (Mumbai, India), unless specified otherwise. Retinoic acid (RA) for differentiation and Silymarin (SilM) for use as standard drug was obtained from Sigma-Aldrich (St. Louis, Missouri, United States). Antibodies for immunohistochemical staining were purchased from Thermo Scientific (anti-vimentin V9, MA5-11883) and Agilent Technologies (EnvisionTM HRP, Dako). Enzyme linked immunosorbent assay (ELISA) kits for rat TNFα (Cat. no. ELR-TNFa-1), rat BDNF (Cat. no. ELR-BDNF-1), rat IL-6 (Cat. no. ELR-IL6-1) and rat MCP-1 (Cat. no. ELR-MCP1-1) were purchased from RayBiotech Life, Inc. (Georgia, USA) and rat HO-1 (Cat. no. orb411280) and rat Nrf2 (Cat. no. orb781880) were purchased from Biorbyt LLC (North Carolina, USA).

### 2.2 Cell culture study

SH-SY5Y (RRID: CVCL_0019), neuroblastoma cells were obtained from National Centre for Cell Science (NCCS, Pune, India) and were provided mycoplasma-free. Cells were cultured in Dulbecco’s Modified Eagle Medium/ Nutrient Mixture F-12 Ham (DMEM/ F12, 1:1 ratio), supplemented with 10% FBS and 1% antibiotic and antimycotic solution, maintained at 37 °C and 5% CO_2_ in a water-jacketed incubator. Cells were subcultured at 70%–80% confluency with adequate medium replenishment. For experimental assays, cells were seeded at a density of 1 × 10^4^ cells per well in 96-well plates and 5 × 10^5^ cells per well in 6-well plates. Cells between passage 25 - 30 were used for the experiments. For neuronal differentiation, cells were treated with 10 mM RA in low-serum medium (1%) for 3 days prior to treatment, following the protocol by (Bustos-Rangel et al., 2023) and validated using morphological changes using ImageJ neurite length analysis (Supplementary Figure S1; Supplementary Table S1). To minimize ethanol evaporation during treatments, plates were sealed with Parafilm (covering ∼75% of the surface, allowing adequate gas exchange) and maintained in a water-jacketed incubator to ensure stable humidity and temperature. Ethanol-treated wells were placed toward the center of the plates, while untreated control wells were positioned at the periphery to minimize differential evaporation. In addition, medium containing ethanol was added to the unused spaces of the culture plates, which has been shown to further reduce evaporation (Rath et al., 2022).

### 2.3 Animal study

Animal experimentation was performed as per standard ethical ARRIVE (Animal research: reporting of *in vivo* Experiments) guidelines and approved (RPCP/IAEC/2023-24/R16) by the Institutional ethical committee of Charotar University of Science and Technology. Eight-to ten-week-old male Wistar rats (weighing 200–250 gm) were procured from SyncBio Research Pvt. Ltd. (Gujarat, India). Before beginning the study, the rats were caged in ambient conditions (25 °C ± 2 °C with relative humidity 55% ± 5%) with two rats per cage. They were provided with a standard chow diet and water *ad libitum*, and allowed to acclimatize for a period of 7 days. They were accommodated in 12 h dark and light cycles throughout the study.

### 2.4 Preparation of GarA stocks for *in vitro* and *in vivo* experiments

Pure GarA was previously extracted, isolated from *G. resinifera* Roth. gum and characterized in the lab (Chadha et al., 2025) was utilized for this study. For the *in vitro* study, the stock solutions (1 mg/mL and 100 μg/mL) were prepared using dimethyl sulfoxide (DMSO) and 1X PBS, ensuring the final DMSO concentration did not exceed 0.1%. For *in vivo* study, GarA and SilM stock solutions (50 mg/mL each) were prepared using 1% carboxy-methyl cellulose (CMC) in sterile water. The stock solutions were prepared under aseptic conditions and autoclaved before use, to ensure sterility.

### 2.5 *In vitro* assays

#### 2.5.1 Cell viability assay using resazurin

Cells were seeded in 96 well plate and allowed to adhere for 20 h, then treated with differentiation media for 3 days. Cells were treated with 10–100 μg/mL concentration of GarA, in increments of 10 μg/mL. After 24 h, the medium was aspirated and cells were washed with 1X PBS and new medium (100 μL) and resazurin was added (0.16 mg/mL, 10 μL) and the plate was incubated at 37 °C with 5% CO_2_ supply for 2 h. Later, the plate was read using a multimode reader for fluorescence at excitation 544 nm and emission 590 nm. No cell and no treatment control wells were kept for baseline correction and calculations of cell viability.

#### 2.5.2 Reactive oxygen species estimation

Cells were seeded and treated similarly for differentiation, followed by GarA treatment (10 μg/mL) with and without the presence of ethanol (500 mM) for 24 h. The concentration of 500 mM ethanol was selected based on preliminary cell viability assays (Supplementary Figure S2), which confirmed that SH-SY5Y cells remain sufficiently viable at this level over the experimental timeframe to allow for mechanistic analyses. Cells were incubated with DCFDA (2′,7′-dichlorodihydrofluorescein diacetate) (10 μM) for staining for 45 min, followed by lysis using Triton X 100 and measurement of fluorescent intensity at excitation 485 nm and emission 535 nm, using a multimode reader.

#### 2.5.3 Observation of nuclear integrity using DAPI staining

Seeding, differentiation and treatment of GarA and ethanol were given similarly as previous section. After 24 h incubation, the cells were fixed using 4% fixing buffer and stained using DAPI (4′,6-diamidino-2-phenylindole) (50 nM) for 15 min. Cells were washed using 1X PBS thrice and fluorescence intensity was measured at 355 nm excitation/ 460 nm emission. Images were captured using a fluorescence microscope using the blue channel.

### 2.6 *In vivo* study

#### 2.6.1 Animal experimentation

After acclimatization, male Wistar rats were randomly divided into five groups: Isocaloric Control (NC), Ethanol-fed (E), Standard drug-SilM (50 mg/kg) (STD), GarA-low dose (50 mg/kg) (GAL) and GarA-high dose (100 mg/kg) (GAH), (Toppo et al., 2017), maintaining a similar average weight in each group. The control group was fed an isocaloric maltodextrin liquid diet, containing equal amounts of calories as compared to the ethanol fed groups, to rule out the variation caused due to caloric differences. While the remaining groups were fed ethanol containing Lieber-DeCarli diet (Supplementary Tables S2-S5). The concentration of maltodextrin and ethanol was increased gradually in 5 days, starting from 1% to 5% on the fifth day. The feeding protocol was continued for 30 days. To mimic normal drinking conditions, the rats were fed with 31.5% maltodextrin and ethanol on 11th and 22nd day (Liu et al., 2023) (Supplementary Table S6). No animals were excluded from the analyses, and the investigators performing further analyses were blinded (using coded identifiers known only to the personnel administering treatment) to the treatment groups.

#### 2.6.2 Brain weight and histopathological observation

Brain samples were collected and weighed. Their size and appearance were noted and compared. Samples were stored in a 10% neutral buffered formalin solution for fixing. Samples were embedded in paraffin wax and washed for 2 h under running water. Blocks were then dehydrated using increasing concentration of isopropyl alcohol (30%–70% – 90%–100%) and deparaffinized using xylene, and impregnated in melted paraffin wax. Thin sections (4–5 μm) of the samples were cut using automated rotary microtome (Leica RM2255). The sections were fixed on poly-L-lysine coated (0.1% w/v in H_2_O) slides and stained using hematoxylin and counterstained with eosin using Gemini AS Automated Slide Stainer (Thermo Scientific). The stained slides were observed under a phase contrast microscope at ×40 magnification (Suvarna et al., 2012).

#### 2.6.3 Immunohistochemical observation

The tissues were fixed, and slides were prepared similarly as in case of HE staining. Sections were de-paraffinized and rehydrated. Autoclave method was utilized for antigen retrieval as per (Bankfalvi et al., 1994), containing antigen retriever buffer of pH 9.0, after a complete cycle of autoclave, the sections were incubated in the buffer for 2 min. Sections were cooled and washed using tris-buffered saline (TBS) pH 7.4, and quenching of endogenous peroxidase was carried out using incubation in 3% H_2_O_2_ for 30 min. The sections were then incubated in anti-vimentin V9 (Thermo scientific, MA5-11883, 1:200) primary antibody. Later, the sections were rinsed with TBS and incubated in EnVision Detection Systems, Peroxidase/DAB, Rabbit/Mouse (Agilent, Dako, Denmark) for 30 min at 25 °C. The sections were counterstained using Mayer’s hematoxylin and mounted. Canine mammary tumor samples were used as positive controls (Wang et al., 2022).

#### 2.6.4 Protein isolation and ELISA

The tissue samples were thawed, and 200 mg samples were taken. The samples were homogenized in 500 μL RIPA buffer (prepared as per Ruiz-Uribe and Bracko, 2020) under ice incubation. After 2 h incubation at - 20 °C, samples were thawed and centrifuged at 12,000 rpm at 4 °C for 20 min. The upper aqueous layer was collected and used for ELISA estimation using specific kits for TNFα, BDNF, heme oxygenase 1 (HO-1), nuclear factor erythroid 2-related factor 2 (Nrf2), IL-6 and MCP-1, following the kits’ protocol.

### 2.7 Gene expression study

SH-SY5Y cells were plated in six well plates and differentiated. After differentiation, cells were treated in four different groups: ethanol (500 mM), GarA (10 μg/mL), ethanol (500 mM) with GarA (10 μg/mL) and no treatment control for 24 h. Brain tissue samples were weighed (100 mg) and homogenized. RNA from both cells and tissue samples was isolated using RNA isoplus (Takara) and cDNA was prepared using kit method (Thermo Verso cDNA synthesis kit). To anticipate the neuroprotective effect of GarA against ethanol in cellular and rodent models, various genes were selected. For *in vitro,* genes for the neurotrophic factor BDNF, pro-inflammatory cytokines and chemokines such as TNFα and MCP-1, and antioxidant genes such as Nrf2 and HO-1, and lastly for the integrity of the BBB, Claudin1 (CLDN) was selected. And for *in vivo*, BDNF, vimentin, PCSK9, HO-1, Nrf2, interleukin 10 (IL-10), MCP-1, TNFα, and CLDN5 were selected. 18S (for *in vitro*) and β-actin (for *in vivo*) were used as the housekeeping genes for the samples. The primers used were designed *in silico* using PrimerBLAST (Ye et al., 2012) and have been published previously (Patel et al., 2021; Aghara et al., 2024; Chadha et al., 2025) (Supplementary Tables S7 and S8). For qPCR, SYBR green master mix and Agilent MxPro3000 were used.

### 2.8 Molecular docking and molecular dynamics (MD) simulations

Three target proteins: BDNF (PDB ID: 1BND)(Robinson et al., 1995), IL-6 (1ALU) (Somers, 1997), and MCP-1 (1DOL) (Lubkowski et al., 1997) were selected for docking analysis. Protein structures were retrieved from the RCSB Protein Data Bank and prepared using Schrödinger’s Protein Preparation Wizard (Madhavi Sastry et al., 2013) (Maestro, v2024-2), including bond order assignment, hydrogen addition, hydrogen-bond optimization (PROPKA, pH 7.4), water removal (>5 Å from hetero groups), and restrained minimization with the OPLS4 force field (Lu et al., 2021) (RMSD cutoff: 0.3 Å). To compare the results of the docking interaction of GarA with the target proteins, docking with known binders of BDNF (4-methyl catechol) (Vetrivel et al., 2012), IL-6 (Pinostrobin chalcone) (Tjiptaningrum et al., 2024) and MCP-1 (Bindarit) (Paccosi et al., 2012), was performed. The three-dimensional structures of GarA and known binders were downloaded from PubChem.

The receptor grid was generated using Glide’s Receptor Grid Generation module (Friesner et al., 2006). The grid was centered on the binding sites of BDNF (Sangeet et al., 2023), IL-6 (Litov et al., 2021) and MCP-1 (Reid et al., 2006) and extended to cover a 20 Å^3^ volume to allow full conformational sampling of the ligand. A van der Waals scaling factor of 1.0 and a partial charge cutoff of 0.25 were used for nonpolar atoms. No positional or hydrogen bond constraints were applied to retain unbiased binding predictions. Docking was carried out using Glide in Extra Precision (XP) mode, which incorporates a more discriminating scoring algorithm that includes terms for hydrophobic enclosure, desolvation penalties, ligand strain energy, and explicit treatment of directional hydrogen bonding. The best-ranked pose based on Glide XP score was selected for post-docking analysis and minimization. Key ligand–protein interactions were visualized using Maestro v12.5 and PyMOL 3.0.2.

To evaluate the stability and dynamic behavior of the protein-ligand complex, molecular dynamics (MD) simulations were performed using the Desmond module (Bowers et al., 2006) in Schrödinger Suite (version 2024-2). The docked complex obtained from XP docking was first imported into the System Builder panel, where it was embedded in an orthorhombic simulation box and solvated using the TIP3P explicit water model (Jorgensen et al., 1983). A buffer distance of 10 Å was maintained between the complex and the edges. The system was neutralized by adding appropriate counterions (Na^+^ or Cl^−^), and 0.15 M NaCl was added to mimic physiological ionic strength. The OPLS4 force field was used for all protein, ligand, and solvent parameters. A series of energy minimizations were performed to eliminate bad contacts, followed by equilibration steps under NVT and NPT ensembles. The production MD simulation was run for 100 ns under NPT ensemble conditions at 300 K temperature and 1.01325 bar pressure, maintained using the Nose-Hoover thermostat and Martyna-Tobias-Klein barostat, respectively (Nosé, 1984; Martyna et al., 1994). A time step of 2 fs was used, and trajectories were saved every 100 ps for analysis. Trajectory analysis included root mean square deviation (RMSD) root mean square fluctuation (RMSF), and ligand–protein interaction profiles over time, using the Simulation Interaction Diagram tool in Maestro. Additionally, the ligand-protein contacts throughout the trajectory were monitored to evaluate the stability and duration of binding interactions over the course of the simulation.

### 2.9 Network pharmacology

To identify probable protein targets of GarA, multiple web-based prediction tools were utilized, including PASS Online (Filimonov et al., 2014), TargetNet (Yao et al., 2016), SwissTargetPrediction (Daina et al., 2019), Similarity Ensemble Approach (SEA) (Keiser et al., 2007), and PharmMapper (Liu et al., 2010). All predicted targets were compiled, and duplicates were removed to generate a non-redundant list of GarA-associated proteins.

Genes associated with ethanol-induced brain damage were retrieved using the keywords “ethanol-induced brain injury” and “ethanol-induced brain damage” from the following databases: NCBI Gene, DisGeNET (Piñero et al., 2019), and GeneCards (Stelzer et al., 2016). All gene lists were standardized and merged, with duplicate entries eliminated.

The intersection between GarA targets and ethanol-related disease genes was determined using Venn diagram analysis (Venny 2.1.0) (Oliveros, 2015) to identify common targets potentially relevant to neuroprotection. These overlapping genes were submitted to the STRING database (Szklarczyk et al., 2023) (confidence score >0.4) to construct a protein–protein interaction (PPI) network. The resulting PPI data were imported into Cytoscape (v3.9.1) (Shannon et al., 2003) for network visualization and analysis, including topological parameter calculation.

To explore the functional significance of the overlapping genes, the top 20 core genes were submitted for Gene Ontology (GO) (biological process, molecular function, cellular component) and Kyoto Encyclopaedia of Genes and Genomes (KEGG) pathway enrichment analyses were performed using ShinyGO v0.77 (Ge et al., 2020). Terms with a p-value <0.05 were considered statistically significant. Enrichment results were visualized as bar plots and bubble charts.

### 2.10 Statistical analysis

Cell culture assays were performed in biological triplicates (n = 3), while animal experimentation was carried out in six biological replicates (n = 6). For histological analyses, multiple fields per animal were quantified, and the average per animal was used as the experimental unit for statistical analysis. Data were expressed as Mean ± SD, where a value of p < 0.05 was considered significant. Statistical analyses were performed using GraphPad Prism 9.1.0 and involved One-way analysis of variance (ANOVA) with either Dunnet’s or Tukey’s HSD post-hoc multiple comparisons test.

## 3 Results

### 3.1 GarA confers neuroprotection in SH-SY5Y cells

**FIGURE 1:**
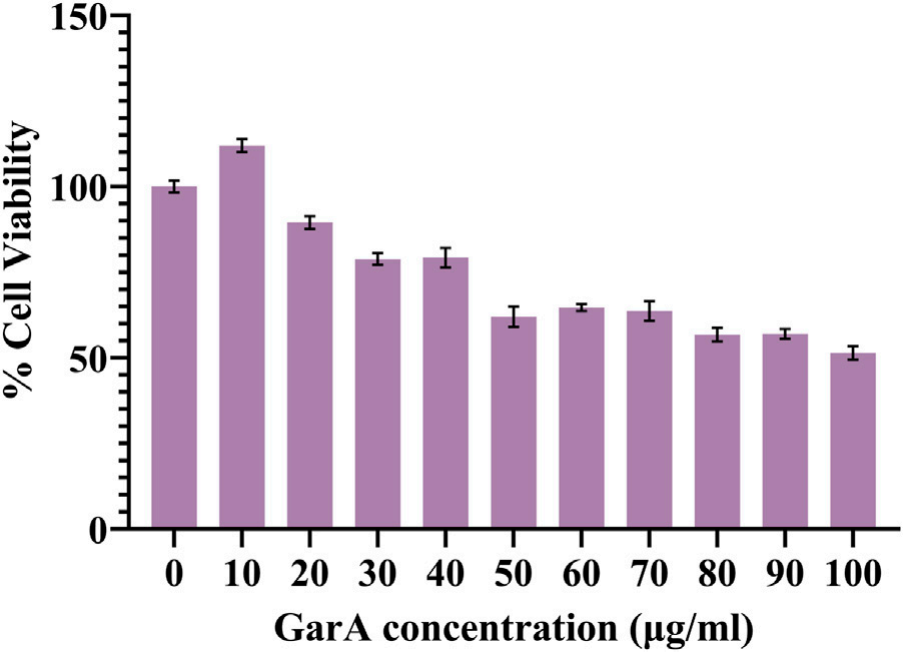
Cell viability of SH-SY5Y cells in presence of GarA (10–100 μg/mL). Data was normalized by control and represented as Mean ± SD. Significance was calculated using One-way ANOVA with Dunnet’s multiple comparisons, where the significance between 0, 10 and 20 μg/mL was p > 0.05, whereas 0, 30 and 40 μg/mL was p < 0.01 and 0 and 50–100 μg/mL was p < 0.0001 (n = 3).

The resazurin assay was performed to evaluate the effect of GarA on the neuroblastoma cell line, SH-SY5Y. Cells showed a dose-dependent decrease in cell viability (Figure 1). Maximum viability was observed at 10 μg/mL GarA, which showed more than 100% viability, showing maintained or slightly enhanced cell viability relative to control. This assay showed that GarA is less toxic till 40 μg/mL, and shows a cell viability above 75%. The lowest viability was observed at a concentration of 100 μg/mL (∼50%).

### 3.2 GarA mitigates ethanol-induced oxidative stress and nuclear damage in SH-SY5Y cells

**FIGURE 2:**
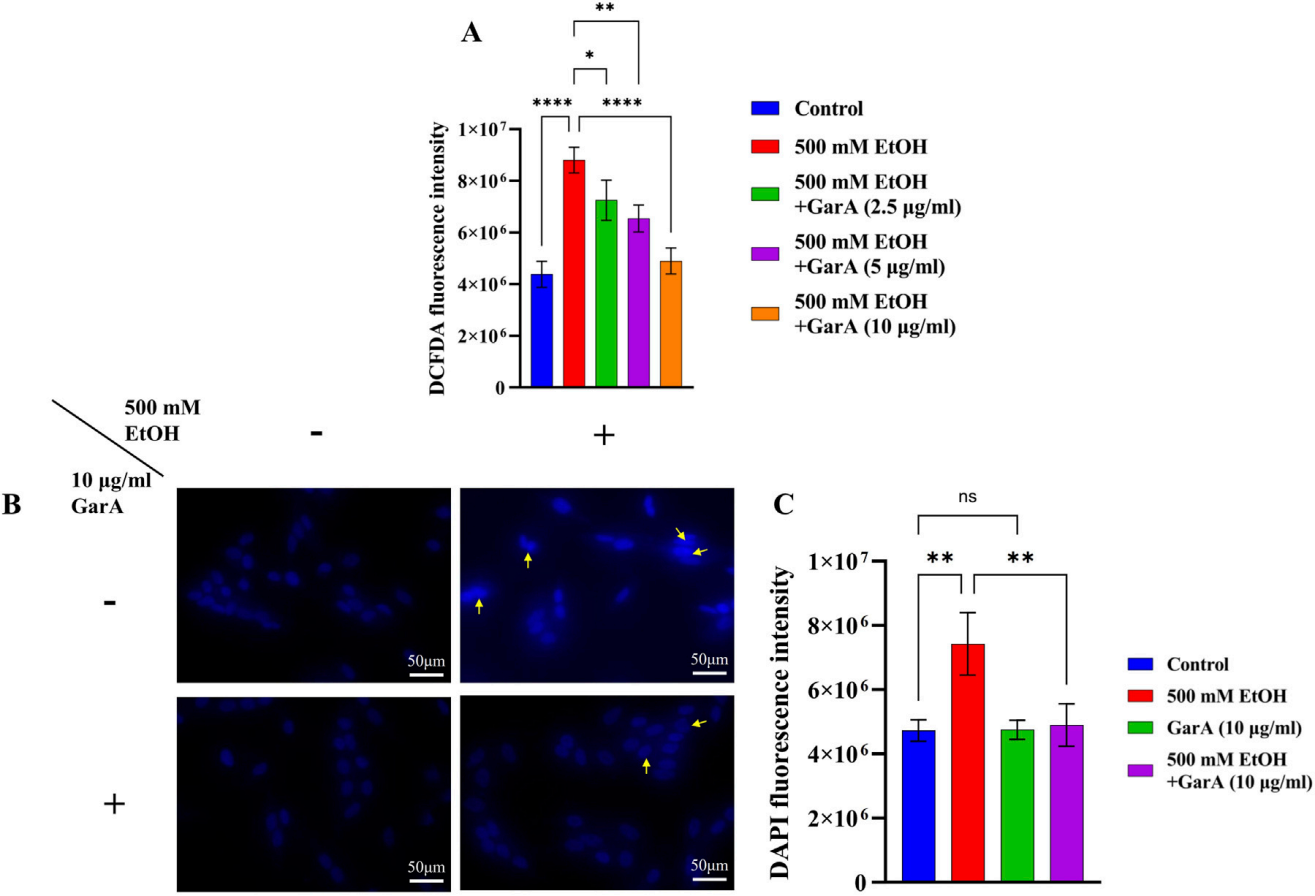
**(A)** DCFDA fluorescence intensity in presence of ethanol (500 mM) and GarA (2.5, 5 and 10 μg/mL); **(B)** Photo-micrographs of SH-SY5Y cells (at ×40 magnification) in presence and absence of ethanol (500 mM) and GarA (10 μg/mL); **(C)** DAPI fluorescence intensity. Values are represented as Mean ± SD and significance was calculated using One-way ANOVA with Dunnet’s multiple comparisons where ^ns^p > 0.05, *p < 0.05, **p < 0.01 and ****p < 0.0001. (n = 3) (Scale bar = 50 μm).

The ROS and nuclear damage caused due to ethanol treatment, and its prevention using co-treatment with GarA was measured using DCFDA and DAPI staining methods. GarA showed protective effect against the ethanol induced oxidative damage, which was evident by a decrease in the DCFDA fluorescence intensity in the co-treatment group, in comparison to the ethanol treatment alone. The cells in the untreated group and the GarA group showed a similar intensity (Figure 2A).

Likewise, the cells showed an uptake of higher amount of DAPI stain in the ethanol treated group. Additionally, the nuclei of that group showed an irregular or fragmented morphology accompanied by nuclear condensation (Figure 2B). The cells in the control, GarA alone and the co-treatment group showed more stable and protected nuclei in comparison to the ethanol group. This trend was observed in the fluorescence intensity of the groups (Figure 2C).

### 3.3 GarA modulates neuroprotective, inflammatory, and barrier-related gene expression *in vitro*

**FIGURE 3:**
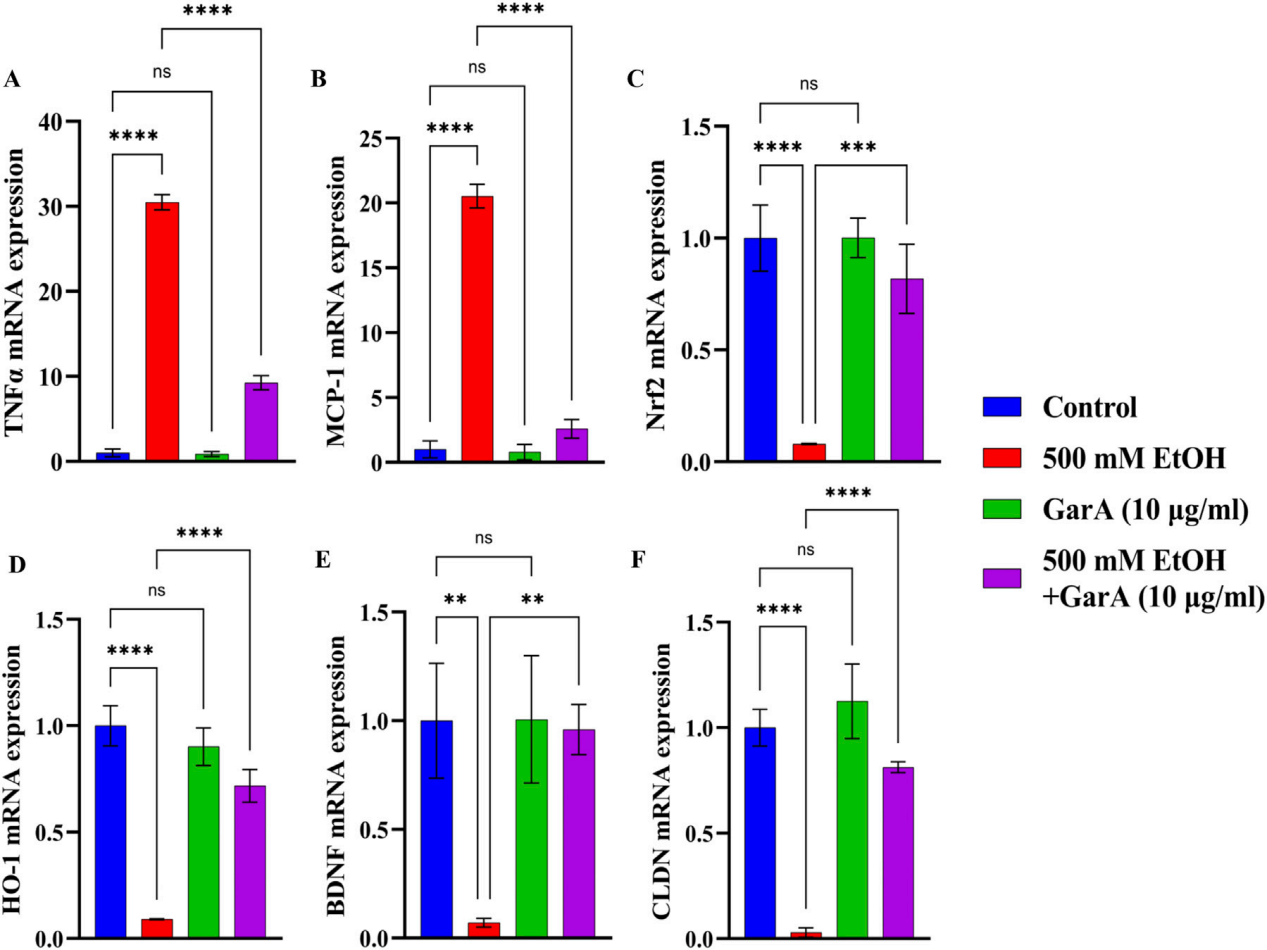
GarA helps reduce inflammation and has an antioxidant and neuroprotective activity in SH-SY5Y cells against ethanol. Gene expression over 18S *in vitro* in different treatment groups showing **(A)**. TNF-α **(B)**. MCP-1 **(C)**. Nrf2 **(D)**. HO-1 **(E)**. BDNF **(F)** CLDN. Values are represented as Mean ± SD and significance was calculated using One-way ANOVA with Dunnet’s multiple comparisons where ^ns^p > 0.05, *p < 0.05, **p < 0.01, ***p < 0.001 and ****p < 0.0001 (n = 3).

The gene expression study in SH-SY5Y cells showed neuroprotective effects of GarA via modulation of various genes. GarA aided in protection of the cells against ethanol induced inflammation, which was evident by a downregulation in the levels of TNFα and MCP-1, respectively, as compared to the ethanol alone treatment (Figures 3A,B). Additionally, GarA showed an increase in the expression of Nrf2 and HO-1, in the co-treatment group, as compared to the ethanol alone treatment group (Figures 3C,D). The expression of BDNF in the GarA (1.00 ± 0.293) and co-treatment groups (0.96 ± 0.115), were comparable to the control group (1.00 ± 0.264) (^ns^p > 0.05), and markedly higher as compared to the ethanol treated cells (0.07 ± 0.020) (Figure 3E). Moreover, the co-treatment with GarA (0.81 ± 0.025) caused an upregulation in the expression of CLDN, in comparison to the ethanol group (0.03 ± 0.022) (Figure 3F).

### 3.4 GarA attenuates ethanol-induced brain injury and inflammation *in vivo*

#### 3.4.1 GarA preserves brain architecture and astrocyte activation status

**FIGURE 4:**
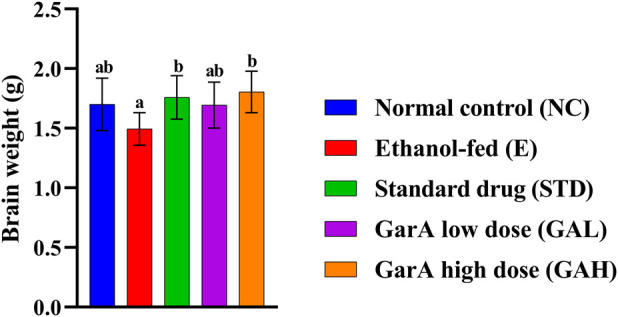
Weight of rodent brain collected after study completion. Values are represented as Mean ± SD and significance was calculated using One-way ANOVA with Tukey’s *post hoc* HSD. Different letters across the treatment groups indicate significant difference p < 0.05 (n = 6).

**FIGURE 5:**
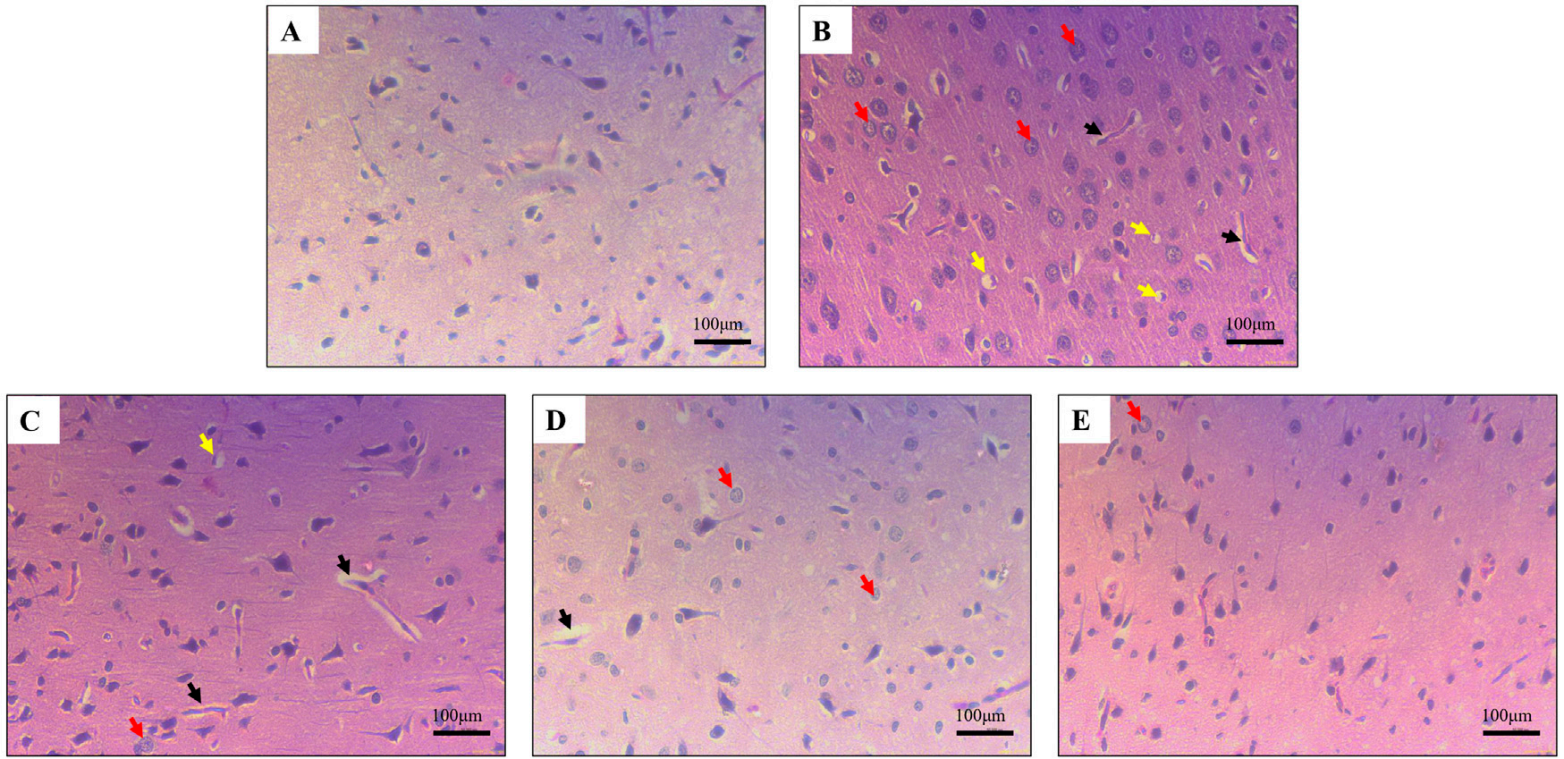
Haematoxylin and eosin staining in rat cerebral cortex showing **(A)** normal morphology in control **(B)** damaged neuronal morphology in ethanol-fed rodents **(C)** and **(D)** slightly improved neuronal morphology in Standard drug treated and GarA low dose (GAL), respectively **(E)**. Improved neuronal tissue and cells in GarA high dose (GAH) treated rodents. Black arrows depict vacuolisation, yellow arrows show pericellular space around necrotic neurons and red arrows show pyknotic nuclei. Images taken at ×40 magnification and scale bar depicts 100 μm.

**FIGURE 6:**
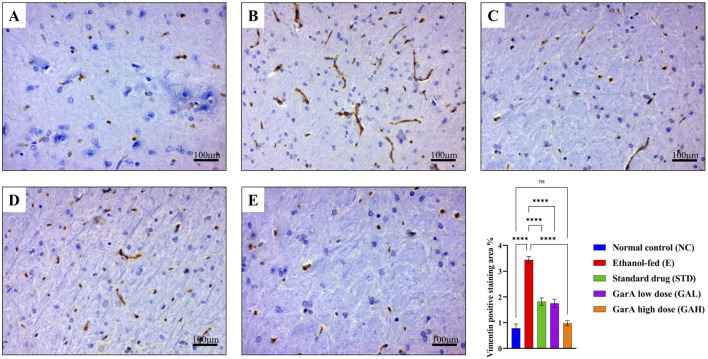
Vimentin immunohistochemistry for staining reactive astrocytes. Photo-micrographs of rat brain cerebral cortex at ×40 magnification stained using anti-vimentin antibody showing **(A)** normal in control **(B)** increased positive staining in ethanol-fed rats and decreased in **(C)** drug treated **(D)** low dose GarA and **(E)** high dose GarA treated rats **(F)**. Graph showing vimentin positive staining area % in different groups (fields/animal). Values are represented as Mean ± SD and significance was calculated using One-way ANOVA with Tukey’s *post hoc* HSD where ^ns^p > 0.05, and ****p < 0.0001 (n = 6, technical replicates).

Chronic alcohol consumption can cause neuronal loss which leads to a decrease in the brain size and weight (Crews, 2008). The rats receiving SilM (STD group), or GarA treatment showed bigger brain sizes, and higher weight (Figure 4) as compared to the disease group. Moreover, the HE staining showed more normal morphology of neurons in the control (100%) and GAH (96.1%) groups, in comparison to the disease group (71.7%) (Figure 5). While the STD and GAL group showed a few anomalies, the morphology appeared improved as compared to the disease group. The neurons in the disease group showed pyknosis (16.6%), formation of vacuoles (5.7%) and pericellular spaces (5.7%) around the necrotic neurons (Ghosh et al., 2021). This trend was decreased in case of STD and GAH groups, where only 2.2% pyknosis was observed in each of those groups. Vimentin is a marker of astrocyte activation and reactive gliosis leading to neurodegeneration (Zhang et al., 2025). Through immunohistochemistry experiment, it was observed, that the brain tissue of the disease group showed increased staining with vimentin, showing higher neuronal activation. This pattern changed, and a decrease in the vimentin uptake was observed in the GAH group (Figure 6). Similarly, the gene expression levels of Vimentin in rat brain tissue showed a similar trend (Figure 8A).

#### 3.4.2 GarA helps in neuroprotection against alcohol induced damage in rats

**FIGURE 7:**
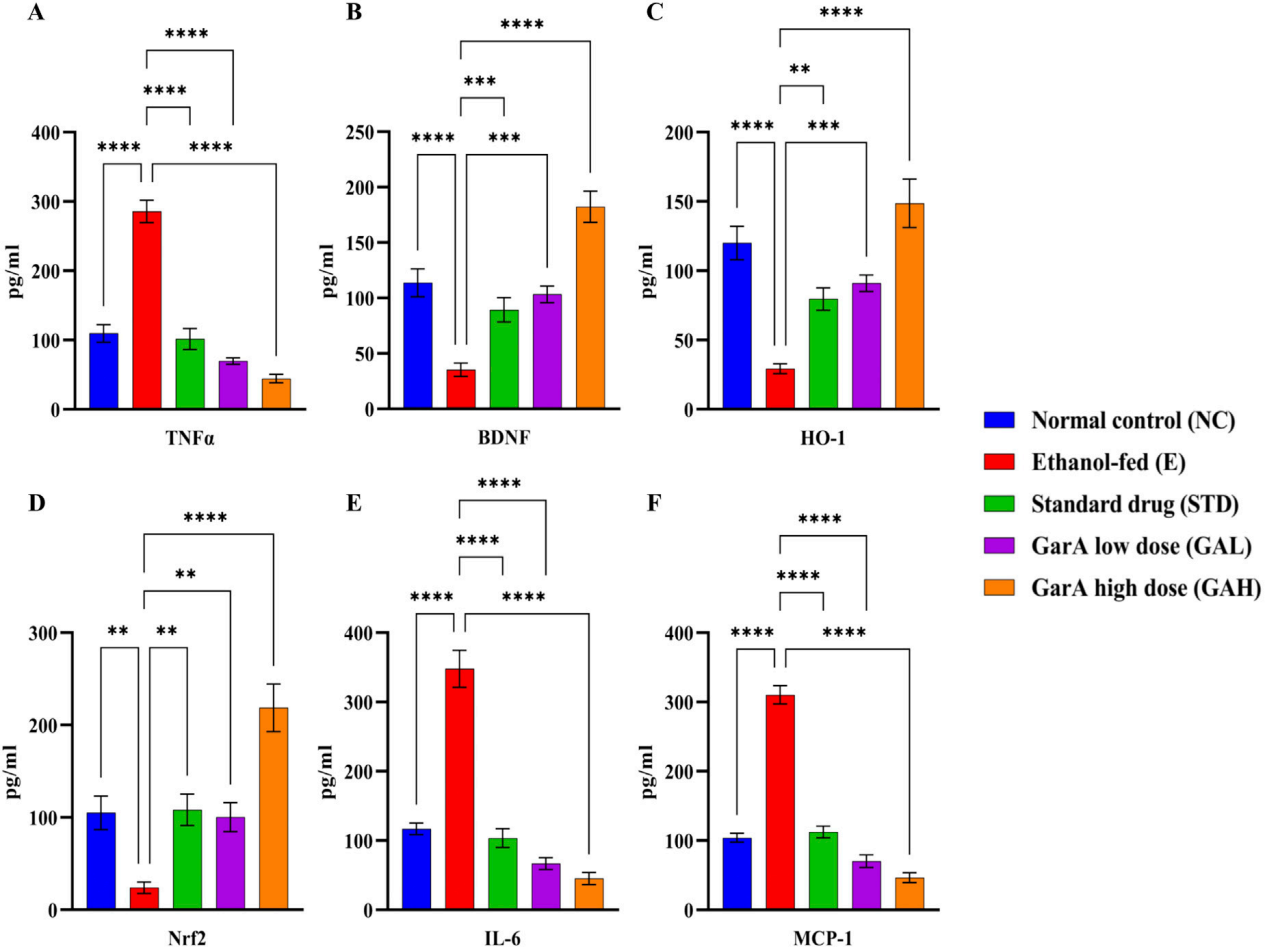
Protein expression profiles of TNFα, BDNF, HO-1, Nrf2, IL-6 and MCP-1 in rat brain tissue samples. Values were represented as Mean ± SD and significance was calculated using One-way ANOVA with Tukey’s multiple comparisons where **p < 0.01, ***p < 0.001 and ****p < 0.0001 (n = 6).

Protein expression in rat brains was estimated using ELISA kits for selected protein targets. TNFα in the diseased group (E), was found to be upregulated 3-folds of the control (NC) rats. While it was observed that the expression decreased in the STD and GarA treated groups. Moreover, the expression of BDNF showed an upregulation in the case of GAH animals, as compared to the NC animals as well. BDNF was diminished in the disease (E) group. Similar trends were observed in the expression of HO-1 and Nrf2 expression profiles, further corroborating the results. Notably, it was observed that for IL-6 and MCP-1, the GAH group showed a drastic reduction in the expression, as compared to all the other groups (Figure 7).

#### 3.4.3 GarA regulates gene expression associated with neuroinflammation and barrier function in rat brain

**FIGURE 8:**
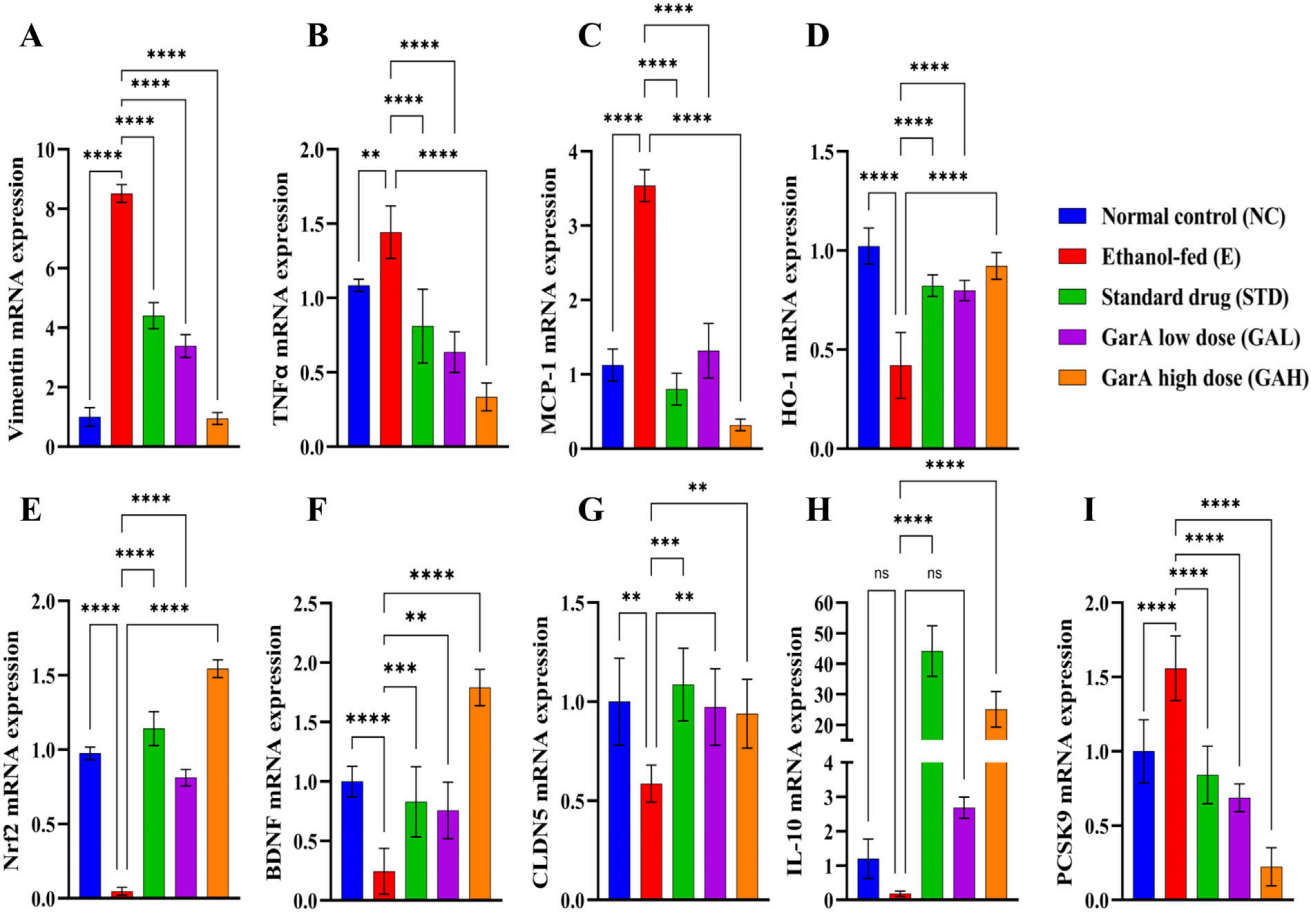
GarA shows neuro-protective effect in rats against alcohol induced damage. Gene expression over β-actin *in vivo* in different treatment groups showing **(A)**. Vimentin **(B)**. TNFα **(C)**. MCP-1 **(D)**. HO-1 **(E)**. Nrf2 **(F)**. BDNF **(G)**. CLDN5 **(H)**. IL-10 **(I)**. PCSK9. Values are represented as Mean ± SD and significance was calculated using One-way ANOVA with Tukey’s multiple comparisons where ^ns^p > 0.05, **p < 0.01, ***p < 0.001 and ****p < 0.0001 (n = 6).

Alcohol consumption causes inflammation, alters brain function and decreases the BBB integrity (Vore and Deak, 2022). Gene expression study carried out using rat brain tissue showed an ethanol-induced increase in TNFα levels in the disease group (1.44 ± 0.176), which was decreased in the GAH group (0.33 ± 0.092), even less than the control group (1.08 ± 0.041) (Figure 8B). Likewise, the chemokine MCP-1 (CCL-2), was upregulated in the disease group (3.54 ± 0.213) and downregulated in the GAH group (0.32 ± 0.077) (Figure 8C). On the contrary, the GAH group showed an upregulation in the expression of HO-1 and Nrf2 levels, as compared to the disease group (Figures 8D,E).

Additionally, the factor for neuronal growth, development and survival, BDNF, was found to be upregulated 2-fold in the GAH group (1.79 ± 0.152) as compared to the control group (1.00 ± 0.012). While the disease group (0.24 ± 0.192) showed an approximate 4-fold decrease in the expression as compared to the control group. Expression for the STD and GAL groups, was comparable to control, but less than the GAH group (Figure 8F).

Furthermore, it was observed that SilM and GarA treatment showed an increase in CLDN5, meaning increased BBB integrity. Contrastingly, the disease group and low dose treatment (GAL) groups showed a decreased expression, hence increased permeability due to the barrier junction dysfunction caused by ethanol (Figure 8G). Notable increase in IL-10 expression in the GAH group was observed, similar to the SilM treated standard group (Figure 8H). Whereas, the PCSK9 expression was decreased drastically in the GAH group and upregulated in the ethanol fed rodents (Figure 8I).

### 3.5 Molecular docking and MD simulations reveal stable complex formation of GarA with BDNF, IL-6, and MCP-1

#### 3.5.1 GarA shows binding in the active site of BDNF, IL-6 and MCP-1

**FIGURE 9:**
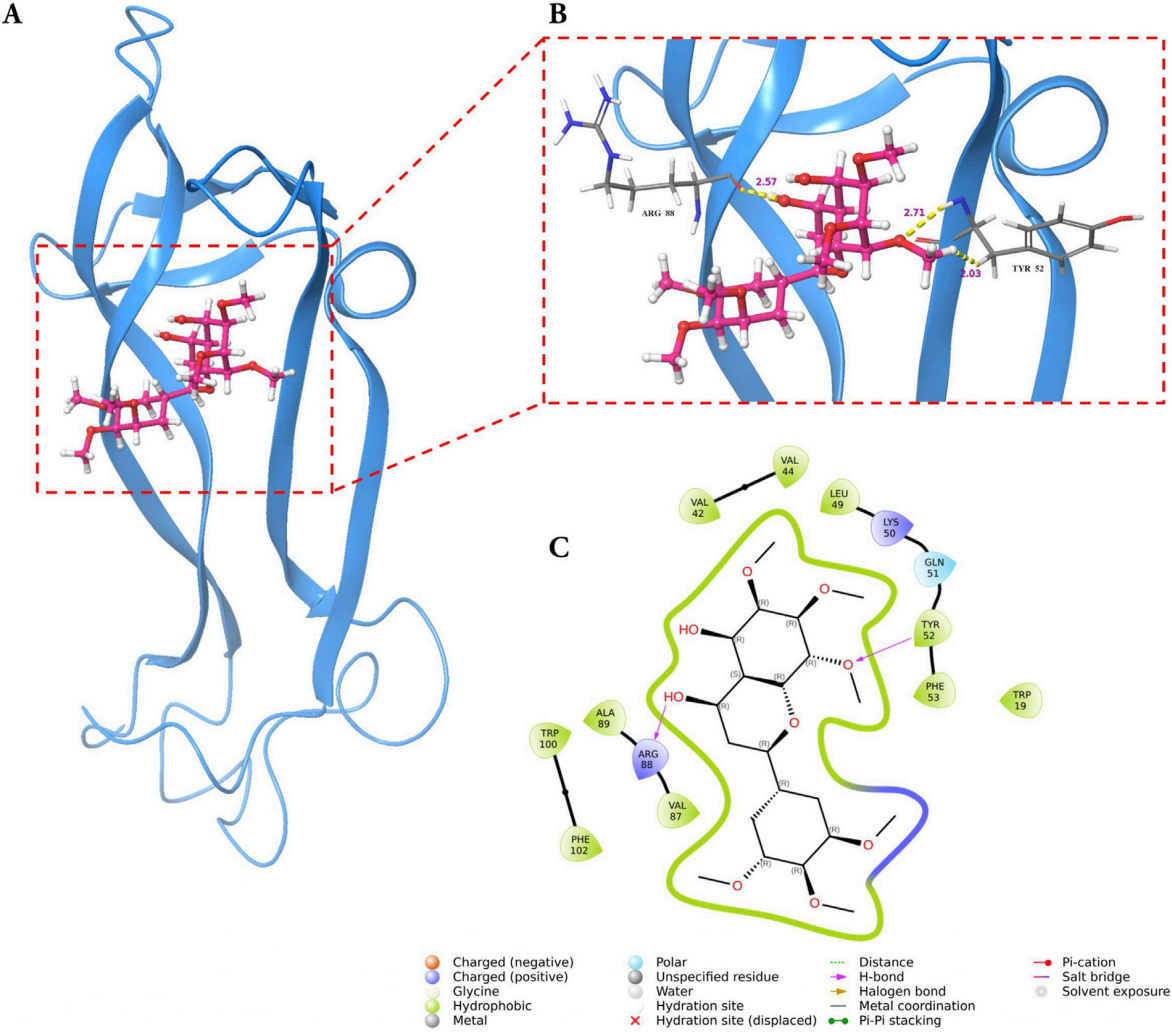
Molecular docking of GarA with BDNF protein where **(A)**. Ribbon representation of the BDNF protein (blue) with the docked GarA (pink) situated in the hydrophobic binding cavity, **(B)**. Zoomed-in view of the binding site showing key hydrogen bonding interactions between the ligand and active site residues including TYR52 and ARG88. Distances (in Å) represent favourable hydrogen bonding geometry, and **(C)**. 2D ligand interaction diagram, illustrating hydrogen bonds, hydrophobic contacts, and surrounding residues.

**FIGURE 10:**
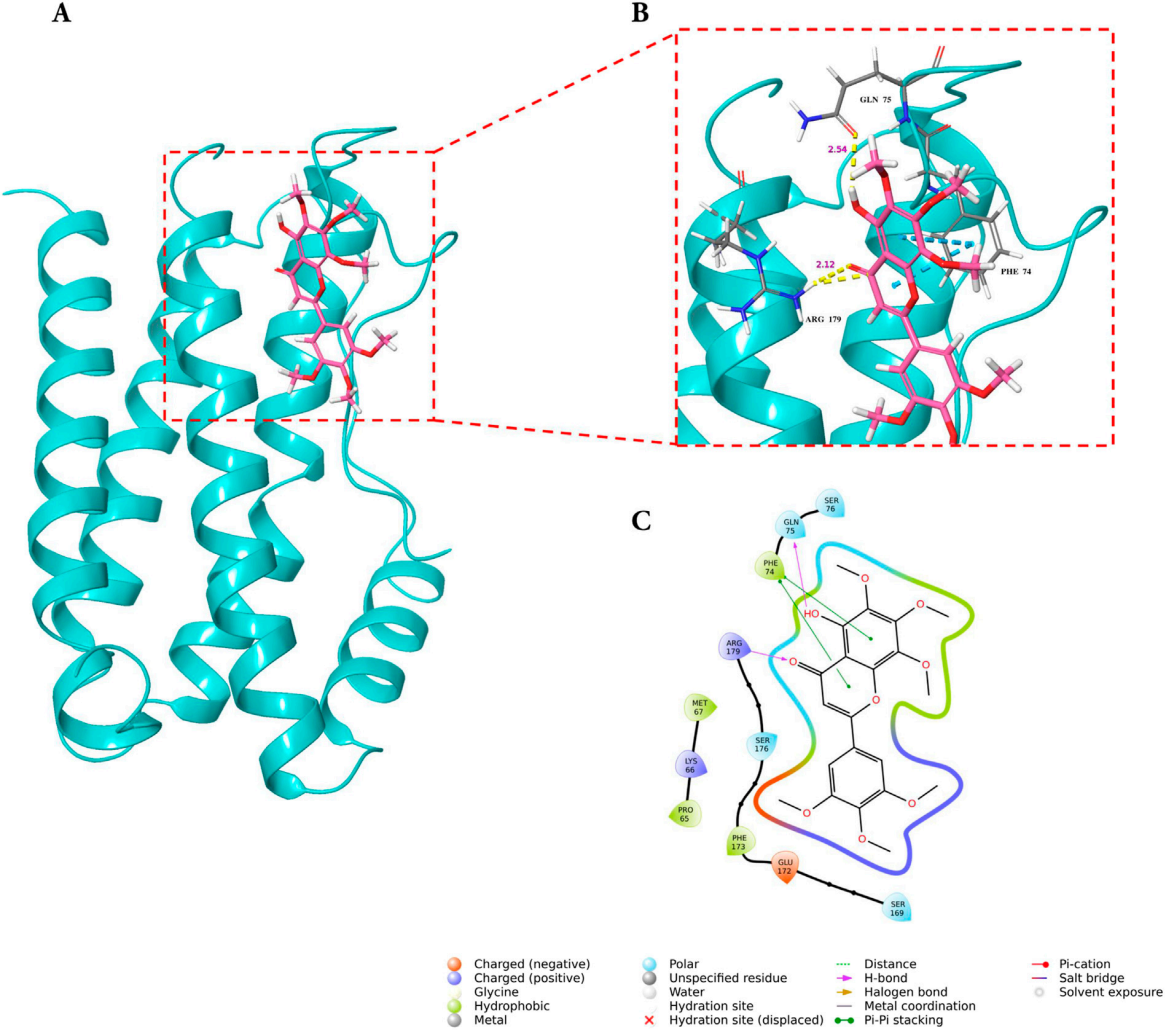
Molecular docking of GarA with IL-6 protein where **(A)**. Ribbon representation of the IL-6 protein (cyan) with the docked GarA (pink) situated in the hydrophobic binding cavity, **(B)** Zoomed-in view of the binding site showing key hydrogen bonding interactions between the ligand and active site residues including PHE74, GLN75, and ARG179. Distances (in Å) represent favourable hydrogen bonding geometry, and **(C)**. 2D ligand interaction diagram, illustrating hydrogen bonds, hydrophobic contacts, and surrounding residues.

**FIGURE 11:**
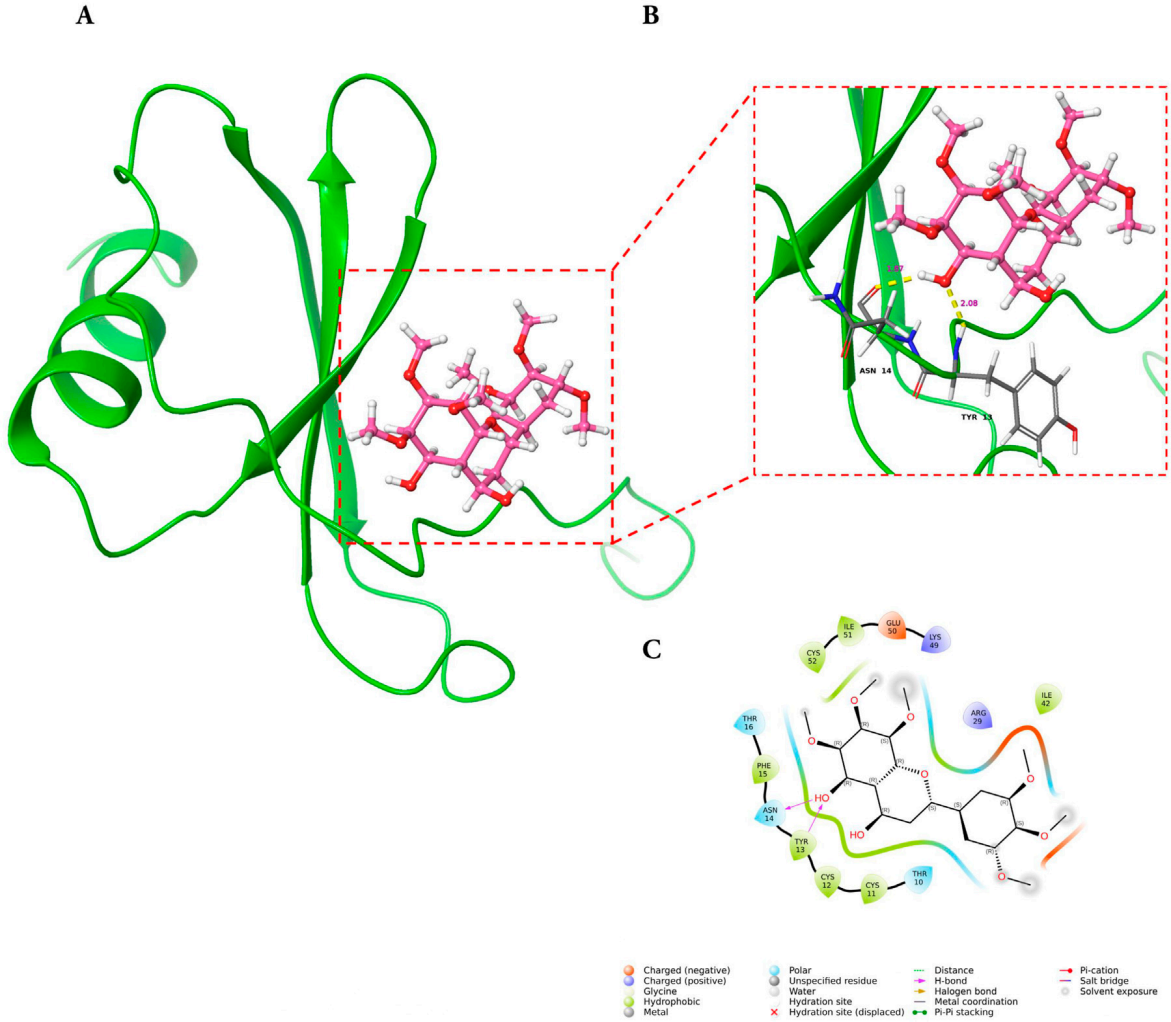
Molecular docking of GarA with MCP-1 protein where **(A)**. Ribbon representation of the MCP-1 protein (green) with the docked GarA (pink) situated in the hydrophobic binding cavity, **(B)** Zoomed-in view of the binding site showing key hydrogen bonding interactions between the ligand and active site residues including TYR13, and ASN14. Distances (in Å) represent favourable hydrogen bonding geometry, and **(C)**. 2D ligand interaction diagram, illustrating hydrogen bonds, hydrophobic contacts, and surrounding residues.

Molecular docking analysis demonstrated that GarA binds effectively in the active sites of BDNF, IL-6, and MCP-1 through a range of stabilizing interactions. In the BDNF–GarA complex (Figure 9), GarA engaged in multiple conventional hydrogen bonds with key residues ARG88 and TYR52 with a binding energy of −4.17 kcal/mol. The complex also had van der Waals and hydrophobic interactions involving TRP19, VAL42, VAL44, LEU49, PHE53, VAL87, ALA89, TRP100 and PHE102. The binding was further stabilized by charged positive interactions with LYS50 and ARG88, and polar interaction with GLN51, highlighting a robust interaction interface. In the IL-6–GarA complex (binding energy = −3.23 kcal/mol) (Figure 10), GarA exhibited strong hydrogen bonding with ARG179 and GLN75, as well as charged negative contact with GLU172 and positive contacts with LYS66 and ARG179. Polar contacts with GLN75, SER76, SER169 and SER176, also contributed to the stable bonding. Notably, π–π stacking with PHE74 and hydrophobic bonding with PRO65, MET67, PHE74 and PHE173, supported additional stabilization. The MCP-1–GarA (Figure 11) complex showed a binding energy of −4.63 kcal/mol supported by hydrogen bonding with TYR13 and ASN14, and hydrophobic interactions with CYS11, CYS12, PHE15, ILE49, ILE51 and CYS52. Polar interactions were also prominent, especially involving THR10, and THR16. These molecular interactions indicate favorable binding conformations across all three protein targets as compared to known ligands, with comparable binding energies and interacting residues (Supplementary Table S9).

#### 3.5.2 MD simulations show stable complex formation of GarA with molecular targets

**FIGURE 12:**
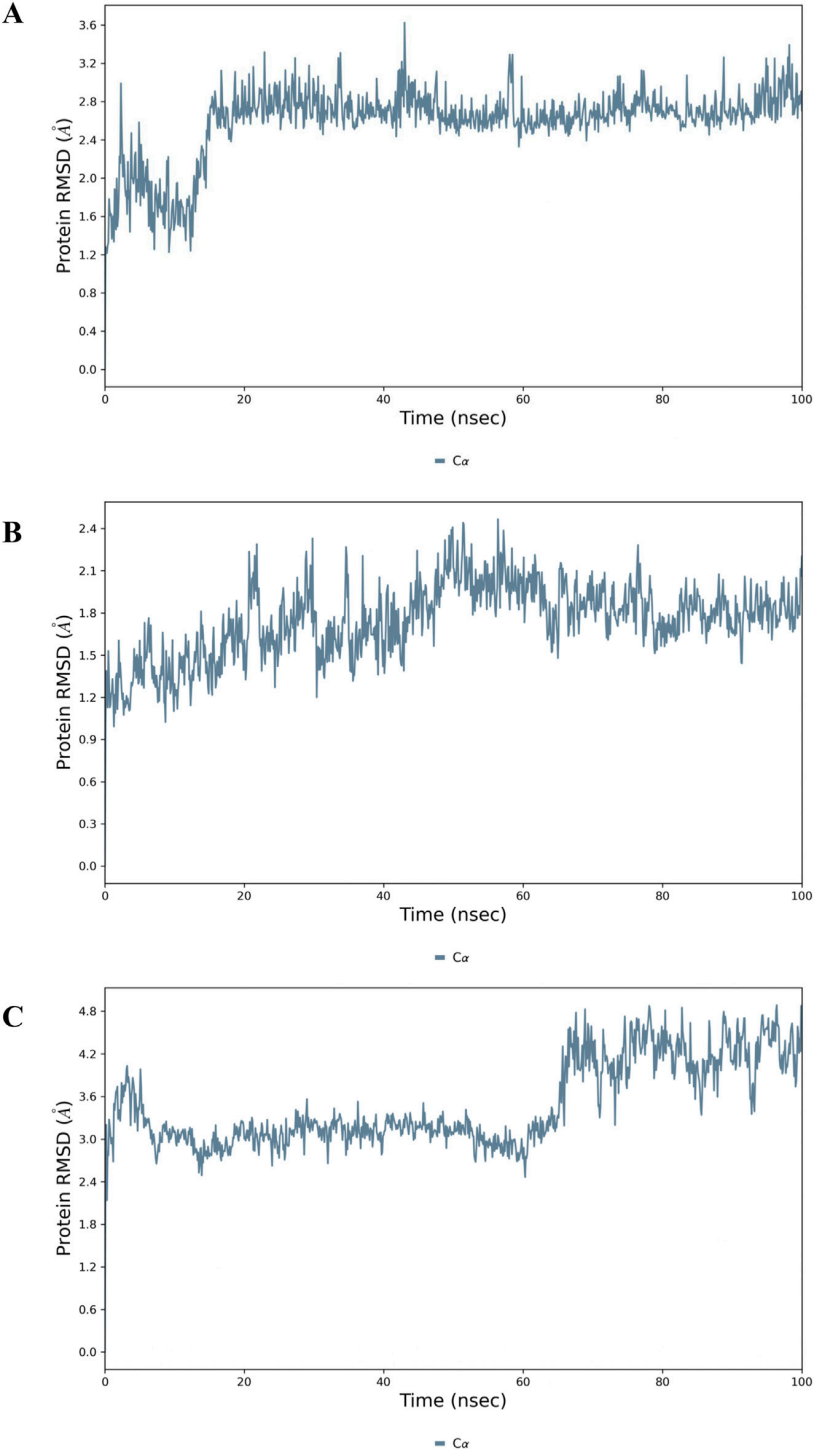
Molecular dynamics simulation analysis of the top docked complexes where Protein RMSD plot showing the stability of the: **(A)** BDNF; **(B)** IL-6 and **(C)** MCP-1 backbone (Cα atoms) over a 100 ns MD simulation.

To further validate the stability of GarA within the protein-ligand complexes, 100 ns molecular dynamics simulations were conducted for BDNF, IL-6 and MCP-1. For the BDNF–GarA complex (Figure 12A), RMSD remained stable around 3.0 Å, and fluctuations were minimal in the structured domains. The ligand RMSD remained under 2.0 Å, with stable hydrogen bonds formed with TYR52 and ARG88. Water bridges with LYS50, TYR52, TYR54 and ARG88 further contributed to ligand affinity, supporting a potential modulatory role of GarA on neurotrophic signalling. The IL-6–GarA complex (Figure 12B) also displayed equilibrium RMSD profiles, with minimal drift and ligand retention within the pocket. Key interactions with MET67, PHE74, GLN75, and ARG179 were observed, involving both hydrophobic contacts and hydrogen bonding. Water-mediated bridges contributed to transient stabilization, reinforcing the interaction profile. The MCP-1–GarA complex (Figure 12C) showed consistent protein backbone RMSD (3.0 Å) with a mild shift at later simulation stages (∼4.5 Å), suggesting local conformational flexibility. Ligand RMSD was stable (<2.5 Å), with residues TYR13 and ASN14 exhibiting persistent hydrogen bonding. Additional hydrophilic and hydrophobic contacts were formed with GLU50, ARG29, and CYS52, stabilizing the ligand within the active site (Supplementary Figures S3-S5 and Supplementary Videos 1-3).

### 3.6 Network pharmacology reveals key targets and pathways of GarA in ethanol-induced brain injury

**FIGURE 13:**
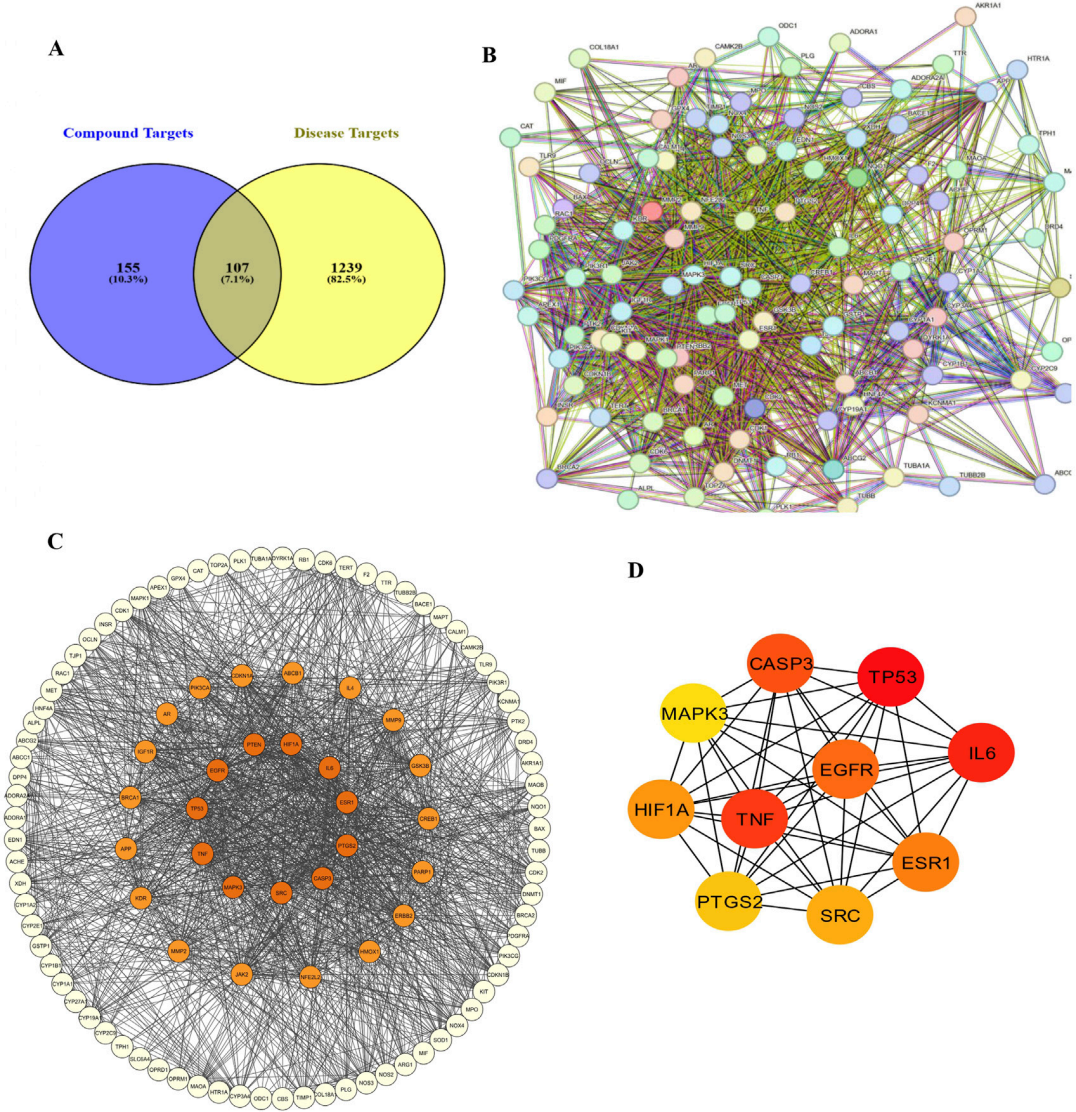
Network pharmacology between Gardenin A and ethanol induced brain damage: **(A)** Venn diagram of Gardenin A target genes and disease-related genes; **(B)** PPI network diagram; **(C)** Network of Gardenin A target genes in Ethanol-induced brain damage; **(D)** Top 10 core target genes of Gardenin A in disease.

**FIGURE 14:**
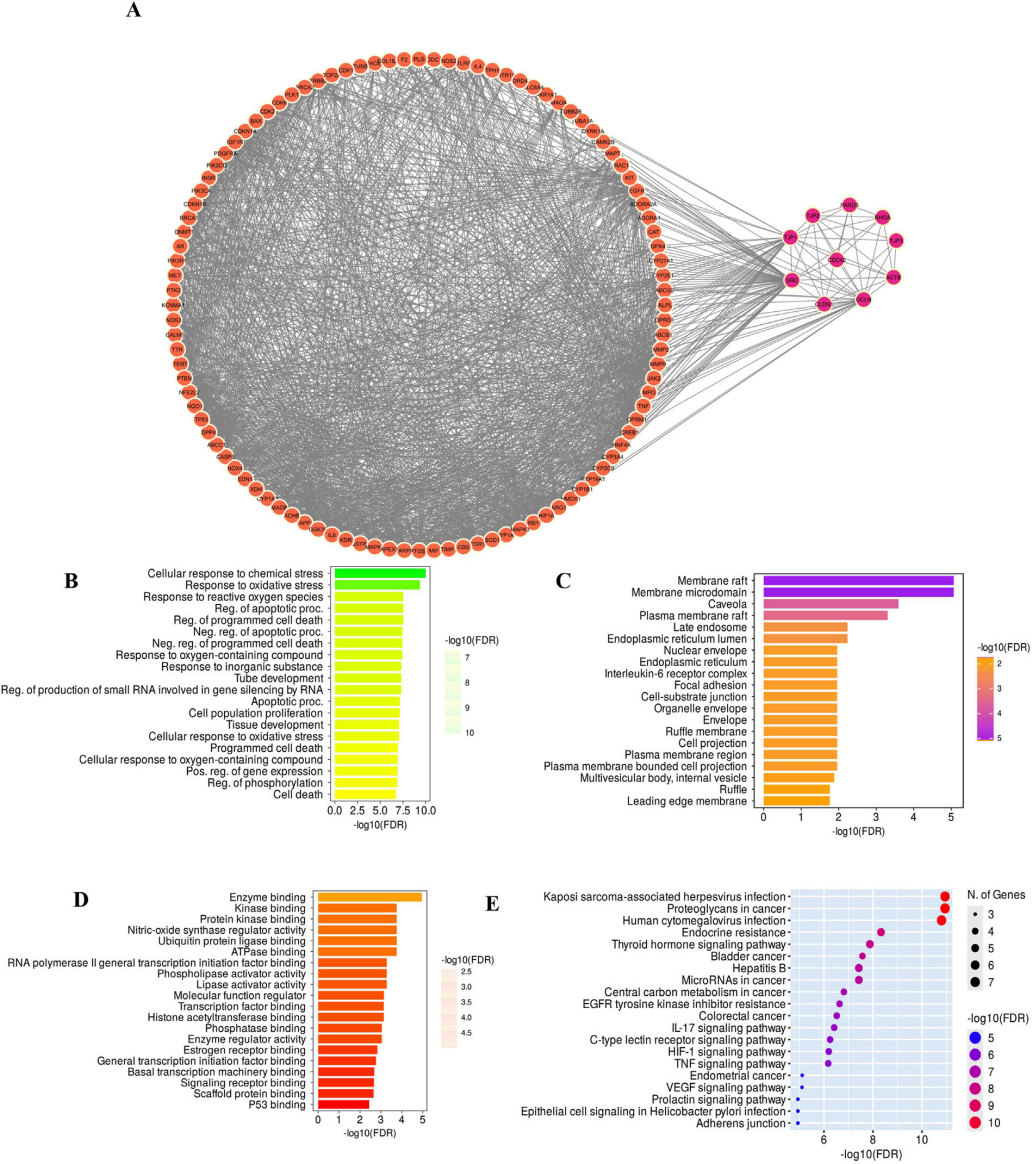
**(A)** Merged Network of GarA target genes and core tight junction genes; **(B)** Graph of the Biological process involved in GO enrichment; **(C)** Cellular components graph of GO enrichment; **(D)** Molecular function graph of GO enrichment; **(E)** KEGG enriched graph for determination of pathways associated with GarA in the treatment of ethanol induced brain damage.

A total of 155 GarA-predicted targets and 1,239 ethanol-associated disease genes were retrieved. After eliminating duplicates, 107 intersecting genes were identified as common to both datasets (Figure 13A). The 107 intersecting genes were input into STRING to generate a PPI network, resulting in a graph with 107 nodes and 1,557 edges, and an average node degree of 29.1, which suggests higher protein interaction (Figure 13B). Genes with darker coloration indicated stronger interaction potential (Figure 13C). Visualization in Cytoscape and analysis via CytoHubba yielded the top 10 core hub genes based on degree centrality (Figure 13D). Similarly, given the role of tight junctions in BBB integrity, a tight junction-specific PPI network was constructed and merged with the GarA-target network. The resulting interaction map highlighted significant overlap, suggesting GarA may influence tight junction-related targets (Figure 14A).

Gene Ontology (GO) analysis of the top core genes revealed enrichment in biological processes such as response to oxidative stress, cytokine activity, and apoptotic regulation (Figures 14B–D). KEGG pathway analysis highlighted the association of GarA with pathways involved in tight junction integrity, TNFα signalling, IL-6, IL-17, HIF-1, and apoptosis (Figure 14E). Among these, the TNFα pathway emerged as a central node, linking oxidative stress, inflammation via IL-6, and BBB dysfunction, which are hallmark features of ethanol-induced neurotoxicity. A few of these predicted targets and pathways such as TNFα, BDNF, and Nrf2/HO-1 signaling, were consistent with the changes observed at the molecular level in the *in vitro* and *in vivo* assays in the current study, thereby reinforcing the biological relevance of the network pharmacology findings.

## 4 Discussion

Chronic ethanol consumption disrupts brain physiology via oxidative stress, glial reactivity, and impaired neurotrophic signaling (Fadda, 1998; Das et al., 2007; Yang and Luo, 2015; Guerri and Pascual, 2019; Li et al., 2020). Sustained ethanol exposure leads to the activation of microglia and astrocytes, resulting in the release of inflammatory cytokines such as TNFα, IL-1β, and MCP-1 (Fernandez-Lizarbe et al., 2009; Zou and Crews, 2012; Pascual et al., 2015; Holloway et al., 2023). These pro-inflammatory mediators exacerbate neuronal damage, disturb synaptic communication, and facilitate neurodegenerative cascades that are implicated in disorders like AD and PD. (Kamal et al., 2020). Despite FDA-approved therapies for alcohol dependence, their impact on systemic health and long-term neuroprotection remains limited (MacKillop et al., 2022). Accordingly, the present study investigates the potential of GarA to prevent alcohol-induced brain damage.

GarA, a polymethoxylated isoflavone previously isolated and characterized (Chadha et al., 2025), was evaluated in this study for its neuroprotective potential. This is the first instance where GarA is investigated for its protective role against ethanol-induced neurotoxicity. In SH-SY5Y cells, GarA significantly enhanced cell viability up to 40 μg/mL and mitigated ethanol-induced oxidative stress by reducing intracellular ROS and stabilizing nuclear morphology. The ROS and DNA damage are caused due to elevated oxidative stress and pro-inflammatory cytokines (Haorah et al., 2005). These effects were consistent with prior reports demonstrating the efficacy of GarA in reducing oxidative damage and inflammation in hepatic and intestinal cell models (Chadha et al., 2025) and EL-4 cells (Yang et al., 2020). In the current study, GarA treatment was associated with decreased pro-inflammatory cytokine levels and an increased expression of antioxidant genes, indicating a protective effect against ethanol-induced cellular damage.

Ethanol consumption in higher amounts leads to reduced brain weight (Das et al., 2007). GarA also exhibited protective effects *in vivo*. Ethanol-fed rodents demonstrated reduced brain weight and significant histoarchitectural alterations, including glial activation and pyknotic nuclei. Although brain concentrations of GarA were not directly measured, its lipophilic nature and previously reported ability to cross the BBB (Hack et al., 2024) suggest that it can access neuronal tissue at biologically relevant levels, supporting the plausibility of the observed *in vivo* neuroprotective effects. This also enables direct modulation of neuronal health and synaptic plasticity. GarA ameliorated neuronal damage and restored normal brain histology. Previously, curcumin showed similar effects in histopathological morphology against rotenone induced PD (Fikry et al., 2022).

Beyond cellular oxidative damage, ethanol also affects neurovascular integrity, particularly the BBB, which is vital for maintaining brain homeostasis. The ROS generated due to ethanol disrupts the BBB due to activation of the myosin light chain kinase (MLCK) and disruption of tight junctions (Haorah et al., 2005; 2007a; 2007b). Here, GarA treatment was associated with preservation of BBB integrity, including modulation of CLDN5 gene expression. It also showed a promising antioxidant effect by causing a rise in the gene and protein levels of HO-1 and Nrf2, while decreasing the pro-inflammatory gene levels. As BBB breakdown allows infiltration of inflammatory mediators and immune cells, it also facilitates the activation of resident glial cells, further amplifying neuroinflammation. While we quantified neuronal damage and glial activation, BBB integrity using protein expression studies was not assessed in the present study. Future work, including quantitative evaluation of tight junction proteins such as CLDN5 will be important to further validate the neuroprotective effects of GarA. Given the upregulation of Nrf2 and HO-1, GarA may exert its antioxidant effects via activation of the Nrf2 signaling pathway, a central mediator of cellular responses to oxidative stress (Qiao et al., 2024). Previous studies have implicated phytochemicals in modulating this pathway (Feng et al., 2022), which could explain the enhanced antioxidant enzyme expression observed in the current model.

Neuroinflammatory reactivity, particularly through TNFα and glial activation, was evident in both gene and protein expression. Immunohistochemical and transcriptional data showed upregulation of vimentin and TNFα in ethanol-exposed rats. GarA significantly reduced their levels, consistent with a potential role in modulating TLR4–NF-κB signaling, as previously implicated in ethanol-mediated neuroinflammation (Fernandez-Lizarbe et al., 2009). A previous study showed alcohol induced neurodegeneration due to binge ethanol consumption in rat model, where vimentin was upregulated due to ethanol-induced cell death (Kelso et al., 2011). Concurrently, the anti-inflammatory cytokine IL-10 was elevated, while PCSK9, a novel neuroinflammatory mediator in the liver–brain axis was downregulated by GarA (Lee et al., 2021). Furthermore, GarA also helped in reducing the protein expression of IL-6 and MCP-1.

Importantly, GarA treatment was associated with restored BDNF expression in both *in vitro* and *in vivo* models, suggesting potential support for synaptic plasticity, learning, and memory. While the upregulation of BDNF is promising, the direct causal linkage between GarA associated-BDNF restoration and neuroprotection remains to be confirmed. Ethanol-induced reductions in BDNF expression are well-documented and contribute to cognitive decline and neurodegeneration (Shafiee et al., 2023). The probable ability of GarA in upregulating BDNF, surpassing control levels, might support its role in neuronal survival and regeneration.

Given the multi-target nature of ethanol-induced neurotoxicity, a network pharmacology analysis was undertaken to predict potential interacting pathways and validate the mechanistic scope of GarA. The integrated molecular docking and MD simulation results support the hypothesis that GarA exhibits a strong multi-target interaction profile relevant to ethanol-induced neurotoxicity. Docking and MD simulation results suggest that GarA may interact with BDNF, IL-6, and MCP-1, indicating potential associations with neurotrophic and inflammatory signaling pathways consistent with experimental observations. These proteins play pivotal roles in alcohol-related brain damage, BDNF is essential for neuronal survival and plasticity, while IL-6 and MCP-1 are central to glial activation and sustained neuroinflammation. Previously, molecular docking with TNFα and GarA has shown high binding affinity with a stable complex formation (Chadha et al., 2025).

The docking affinity of GarA across all three targets, combined with the observed conformational stability during MD simulations, indicates its probable potential to maintain prolonged interaction under physiological conditions. The low ligand RMSD values and stable protein backbones in the simulations further suggest that GarA can occupy and persist within the active sites without significant disruption, enhancing its likelihood of effective biological modulation. MD simulations were performed as single 100-ns trajectories due to computational constraints; while replicates would provide error estimates, the detailed parameters, RMSD/RMSF profiles, and deposited files ensure reproducibility and offer meaningful insights into complex stability. Further, *in vitro* protein assays may help clarify the binding modes of GarA with these targets.

**FIGURE 15:**
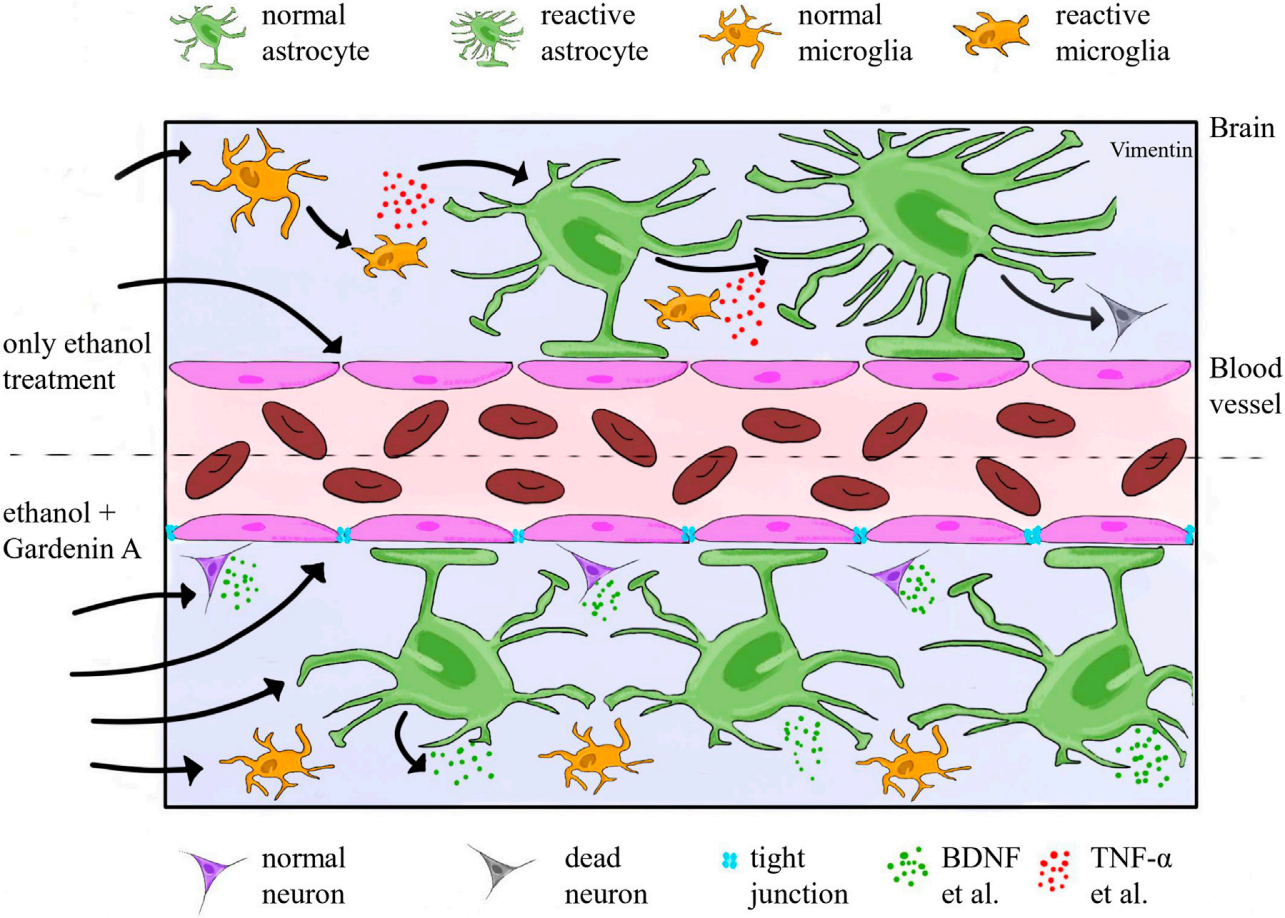
Schematic diagram showing cellular and molecular mechanisms occurring in the brain and BBB in the state of intoxication (upper half) and GarA provided as a drug along with ethanol (lower half), separated by dashed line. Ethanol exposure is associated with glial activation, increased pro-inflammatory cytokines such as TNFα, IL-6 and potential disruption of BBB. GarA is shown to be linked with modulation of neuronal and glial responses, including factors such as BDNF, Nrf2, HO-1 and IL-10. Image drawn using SketchBook app v6.0.4.

These findings align well with the results of network pharmacology, which highlighted TNFα, IL-6, and related pathways as key modulatory hubs in ethanol-induced damage. By simultaneously targeting proteins involved in inflammatory signaling and neuroprotection, GarA may offer a synergistic mode of action, suppress pro-inflammatory cascades while preserving or enhancing neurotrophic support (Figure 15). These associative findings support further investigation into GarA as a potential therapeutic candidate in alcohol-related neurodegenerative conditions.

The current study combined *in vitro*, *in vivo*, and *in silico* approaches to generate converging evidence that GarA is associated with modulation of neuroinflammatory and neuroprotective pathways, including TNFα, BDNF, and Nrf2/HO-1. Although direct perturbation studies with pathway-specific inhibitors or genetic knockdowns were not included, the consistency of findings across experimental systems provides strong support for the involvement of these signaling mechanisms. Future investigations incorporating such targeted interventions would help to further delineate mechanistic specificity. Further, given that sex-specific differences in neuroinflammation and neurodegeneration are increasingly recognized, inclusion of both sexes in future studies will be essential to establish the broader relevance of these findings. From a translational perspective, the doses of GarA used in rodents (50 and 100 mg/kg) were guided by prior safety and efficacy reports; however, these doses cannot be directly extrapolated to humans due to interspecies differences in metabolism, pharmacokinetics, and bioavailability. Collection of pharmacokinetic and pharmacodynamic data, together with dose-scaling studies, are required to evaluate clinical feasibility. Additionally, complementary models and longer-term experimental paradigms may be necessary to further validate and extend the translational relevance of these findings.

## Data Availability

The original contributions presented in the study are included in the article/Supplementary Material, further inquiries can be directed to the corresponding author.

## Supplementary material

SUPPLEMENTARY VIDEO 1 Simulation video file BDNF-GarA.

SUPPLEMENTARY VIDEO 2 Simulation video file IL6-GarA.

SUPPLEMENTARY VIDEO 3 Simulation video file MCP1-GarA.
