# Supplementary material for: Modulation of TNFα-driven neuroinflammation by Gardenin A: insights from in vitro, in vivo, and in silico studies
Source: Front Pharmacol. 2025 Nov 10;16:1681403. doi: 10.3389/fphar.2025.1681403 (PMC12640973; doi:10.3389/fphar.2025.1681403)
Supplement: Supplementary file 4 [file DataSheet1.docx]

Supplementary Material 1

# SH-SY5Y differentiation validation


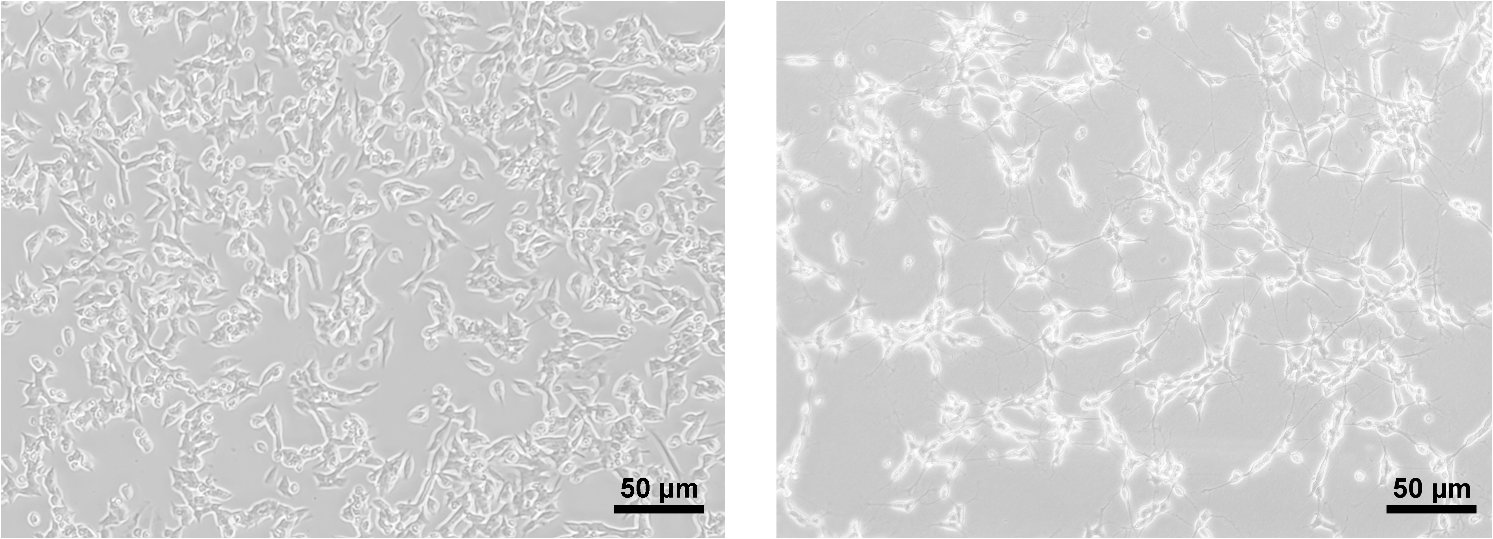


**Supplementary Figure 1.** SH-SY5Y cell; Left: Undifferentiated cells showing immature cell morphology (absence of neurites); Right: Differentiated cells showing neuronal morphology with distinct neurite growth; Scale bar 50 μm; Magnification 40X.

Supplementary Table 1: Neurite length of differentiated SH-SY5Y cells measured using ImageJ

| Sr. no. | Length | Sr. no. | Length (μm) |
| --- | --- | --- | --- |
| 1 | 24.817 | 7 | 21.645 |
| 2 | 47.395 | 8 | 22.858 |
| 3 | 32.04 | 9 | 40.244 |
| 4 | 35.489 | 10 | 26.028 |
| 5 | 19.199 | Mean | 29.379 |
| 6 | 24.076 | SD | 9.148 |

# Standardization of ethanol concentration for experiments


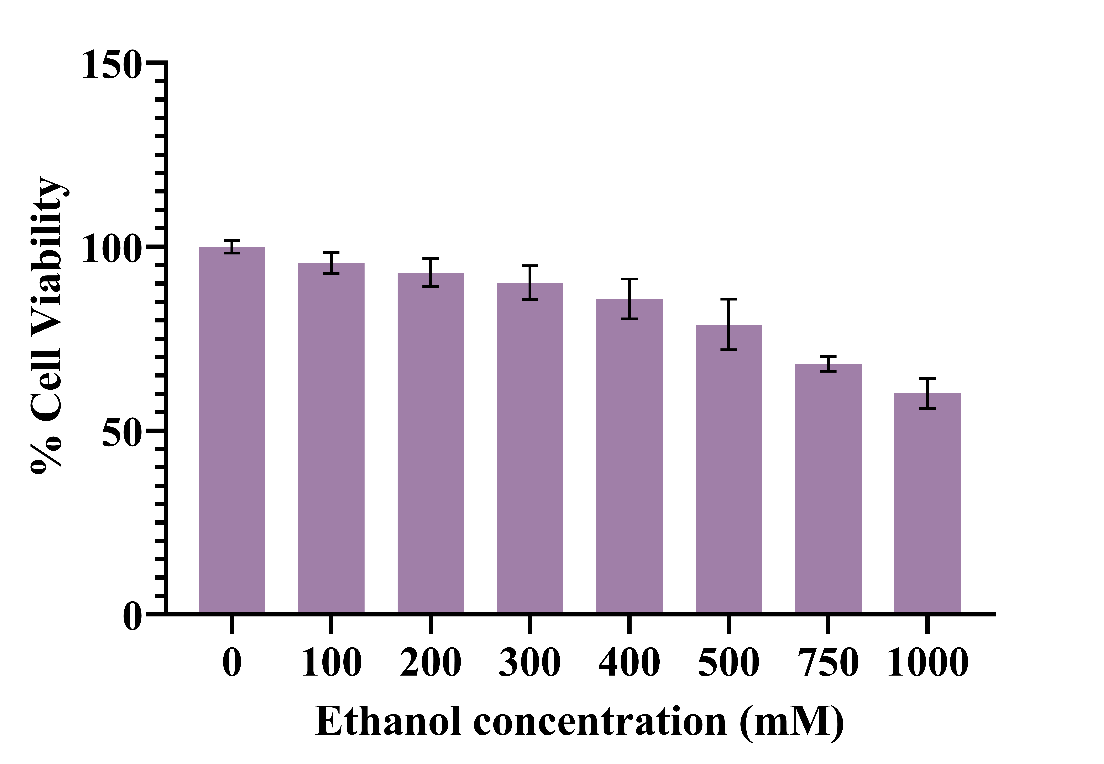


Supplementary Figure 2: Cell viability (%) of SH-SY5Y cells in presence of ethanol (100 - 1000 mM)

## Lieber DeCarli diet preparation

**Supplementary Table 2:** Composition for Lieber de Carli liquid diet

| Sr. no. | Ingredient | Amount (gm) / litre |
| --- | --- | --- |
| 1. | Casein | 41.40 |
| 2. | Methionine | 0.30 |
| 3. | Cystine | 0.50 |
| 4. | Cellulose | 10.00 |
| 5. | Corn oil | 39.60 |
| 6. | Choline | 0.53 |
| 7. | Xanthan gum | 3.00 |
| 8. | Mineral mix | 8.75 |
| 9. | Vitamin mix | 2.50 |

**Supplementary Table 3:** Composition for Vitamin mix

| Sr. no. | Vitamin | Amount / litre |
| --- | --- | --- |
| 1. | Thiamine hydrochloride | 6.0 mg |
| 2. | Riboflavin | 6.0 mg |
| 3. | Niacin | 30.0 mg |
| 4. | Pyridoxine hydrochloride | 7.0 mg |
| 5. | Calcium pantothenate | 16.0 mg |
| 6. | Folic acid | 2.0 mg |
| 7. | Biotin | 0.2 mg |
| 8. | 0.1% cyanocobalamine | 10.0 mg |
| 9. | Menadione sodiumbissulphite | 0.8 mg |
| 10. | Vitamin E acetate | 24 IU |
| 11. | Vitamin D3 | 400 IU |
| 12. | Inositol | 100.0 mg |
| 13. | P-amino benzoic acid | 50.0 mg |

**Supplementary** **Table 4:** Composition for Mineral mix

| Sr. no. | Mineral | Amount (mg) / litre |
| --- | --- | --- |
| 1. | Calcium carbonate, dibasic | 500.00 |
| 2. | Potassium citrate H_2_0 | 220.00 |
| 3. | Sodium chloride | 74.00 |
| 4. | Potassium sulphate | 52.00 |
| 5. | Magnesium oxide | 24.00 |
| 6. | Ferrous sulphate 7H_2_O | 4.95 |
| 7. | Zinc carbonate | 1.60 |
| 8. | Manganous sulphate H_2_0 | 4.60 |
| 9. | Cupric carbonate | 0.30 |
| 10. | Potassium iodate | 0.01 |
| 11. | Sodium selenite | 0.01 |
| 12. | Chromium potassium sulphate 12H_2_O | 0.55 |
| 13. | Sodium fluoride | 0.06 |
| 14. | Sucrose | 117.90 |

**Supplementary Table 5:** Composition for ethanol and maltodextrin for 1000 ml diet

| Group | Component | Amount in 1000 ml diet | | | | | |
| --- | --- | --- | --- | --- | --- | --- | --- |
|  |  | 1% | 2% | 3% | 4% | 5% | 31.5% |
| Ethanol fed (E) | Ethanol (95% v/v) (ml) | 10.5 | 21.05 | 31.6 | 42.1 | 52.6 | 331.6 |
|  | Maltodextrin (gm) | 5.12 | 10.24 | 15.36 | 20.48 | 25.6 | 161.28 |
| Isocaloric Control (NC) | Maltodextrin (gm) | 23.04 | 46.08 | 69.12 | 92.16 | 115.2 | 725.76 |

## Diet and dosing timeline for animal study

Supplementary Table 6: Animal dosing and feeding timeline

| Day | Isocaloric group | Disease control | Standard drug | Test drug - Low dose | Test drug - High dose |
| --- | --- | --- | --- | --- | --- |
| Day 1-7 | Acclimatization with food pellet and Water (*ad libitum*) | | | | |
| Day 8-12 | Isocaloric Maltodextrin + Diet mix | Lieber de Carli liquid diet with 1-5% ethanol increasing from day 1 to 5 + Diet mix | Lieber de Carli liquid diet with 1-5% ethanol increasing from day 1 to 5 + Diet mix | Lieber de Carli liquid diet with 1-5% ethanol increasing from day 1 to 5 + Diet mix | Lieber de Carli liquid diet with 1-5% ethanol increasing from day 1 to 5 + Diet mix |
| Day 13-17 | Isocaloric Maltodextrin + Diet mix | Lieber de Carli liquid diet with 5% ethanol + Diet mix | Lieber de Carli liquid diet with 5% ethanol + Silymarin (50 mg/kg) | Lieber de Carli liquid diet with 5% ethanol + Gardenin A (50 mg/kg) | Lieber de Carli liquid diet with 5% ethanol + Gardenin A (100 mg/kg) |
| Day 18 | Isocaloric Maltodextrin + Diet mix | Lieber de Carli liquid diet with 31.5% ethanol + Diet mix | Lieber de Carli liquid diet with 31.5% ethanol + Silymarin (50 mg/kg) | Lieber de Carli liquid diet with 31.5% ethanol + Gardenin A (50 mg/kg) | Lieber de Carli liquid diet with 31.5% ethanol + Gardenin A (100 mg/kg) |
| Day 19-28 | Isocaloric Maltodextrin + Diet mix | Lieber de Carli liquid diet with 5% ethanol + Diet mix | Lieber de Carli liquid diet with 5% ethanol + Silymarin (50 mg/kg) | Lieber de Carli liquid diet with 5% ethanol + Gardenin A (50 mg/kg) | Lieber de Carli liquid diet with 5% ethanol + Gardenin A (100 mg/kg) |
| Day 29 | Isocaloric Maltodextrin + Diet mix | Lieber de Carli liquid diet with 31.5% ethanol + Diet mix | Lieber de Carli liquid diet with 31.5% ethanol + Silymarin (50 mg/kg) | Lieber de Carli liquid diet with 31.5% ethanol + Gardenin A (50 mg/kg) | Lieber de Carli liquid diet with 31.5% ethanol + Gardenin A (100 mg/kg) |
| Day 30 | Termination of study. (after fasting period of 12 hours)  Blood was collected from retro orbital plexus, and the animals were sacrificed as per CPCSEA recommended method. Tissues were collected from the animals. | | | | |

*Dosing of SilM and GarA was given via Oral gavaging

## Primer sequences for gene expression study

Supplementary Table 7: Gene sequences for *Homo sapiens* primers

| Sr. no. | Gene | Forward primer | Reverse primer |
| --- | --- | --- | --- |
| 1. | 18S | GATGGTAGTCGCCGTGCC | GCCTGCTGCCTTCCTTGG |
| 2. | BDNF | CATCCGAGGACAAGGTGGCTTG | GCCGAACTTTCTGGTCCTCATC |
| 3. | MCP-1 | TCGCTCAGCCAGATGCAAT | TGGCCACAATGGTCTTGAAG |
| 4. | TNFα | CTCTTCTGCCTGCTGCACTTG | ATGGGCTACAGCTTGTCACTC |
| 5. | HO-1 | AAGCCGAGAATGCTGAGTTCA | CGGGTGTAGATATGGTACAAGGA |
| 6. | Nrf2 | GAGAGCCCAGTCTTCATTGC | TGCTCAATGTCCTGTTGCAT |
| 7. | CLDN | CCAGTCAATGCCAGGTACGAAT | TTGGTGTTGGGTAAGAGGTTGTT |

Supplementary Table 8: Gene sequences for *Rattus norvegicus* primers

| Sr. no. | Gene | Forward primer | Reverse primer |
| --- | --- | --- | --- |
| 1. | β-actin | TCTGTGTGGATTGGTGGCTCTA | CTGCTTGCTGATCCACATCTG |
| 2. | BDNF | GAGCCGAGCTCATCTTTGCC | CACTGCGCCCTAGCACAAAA |
| 3. | PCSK9 | AACTTGGCGTCTCATCCTGG | AGCTGGCCAAAGCCAACTAT |
| 4. | TNFα | CAAGGAGGAGAAGTTCCCAA | CTCTGCTTGGTGGTTTGCTA |
| 5. | HO-1 | AGAGTTTCCGCCTCCAACCA | CGGGACTGGGCTAGTTCAGG |
| 6. | Nrf2 | GCAACTCCAGAAGGAACAGG | AGGCATCTTGTTTGGGAATG |
| 7. | Vimentin | TGAGATCGCCACCTACAGGA | GAGTGGGTGTCAACCAGAGG |
| 8. | CLDN5 | GAACTACGTCTAAGGGCGGG | AATTCAGCGGTGGTCGTCAT |
| 9. | MCP-1 | GGTCTCTGTCACGCTTCTG | TCTTGCCAGTGAATGAGTAGC |
| 10. | IL-10 | GCCTTGTCAGAAATGATCA | TGATCATTTCTGACAAGGC |

# Molecular docking interactions

Supplementary Table 9: Molecular docking interactions of GarA and known ligands with selected macromolecules

| Sr. no. | Macromolecule | Interaction with GarA | | Interaction with known ligand | |
| --- | --- | --- | --- | --- | --- |
|  |  | Docking score  (kcal/mol) | Residues forming H bonds | Docking score (kcal/mol) | Residues forming H bonds |
| 1. | BDNF | -4.17 | TYR52, ARG88 | -5.68 | TYR52 |
| 2. | IL-6 | -3.23 | GLN75, ARG179 | -4.75 | PHE74 |
| 3. | MCP-1 | -4.63 | TYR13, ASN14 | -3.87 | CYS52 |

# Ligand-Protein interaction stability

- 1. **BDNF-GarA interaction**


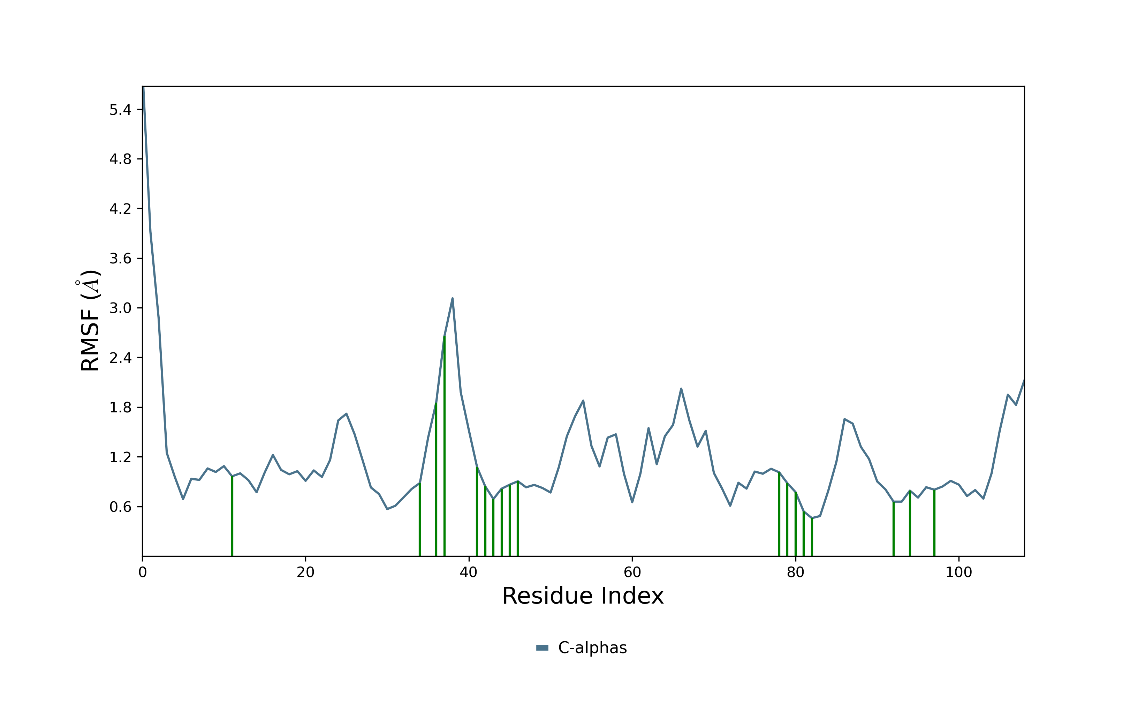


**A**


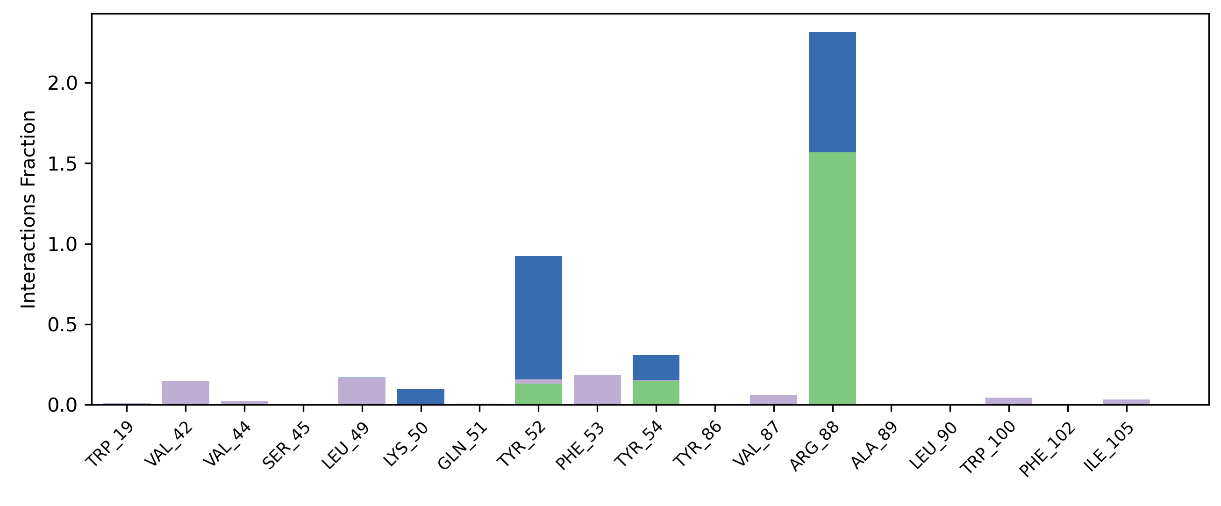


**B**

Supplementary Figure 3: Graphs showing molecular complex stability of GarA with BDNF: A. RMSF and B. Interaction residues

- 1. **IL-6-GarA interaction**


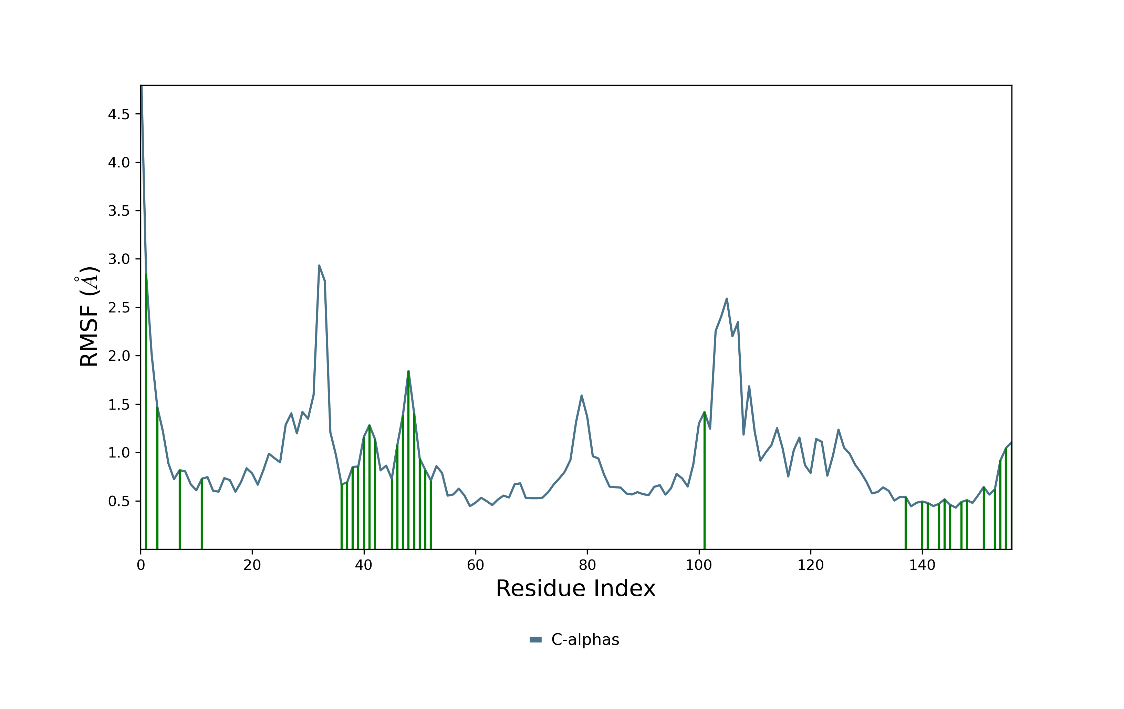


**A**

**B**


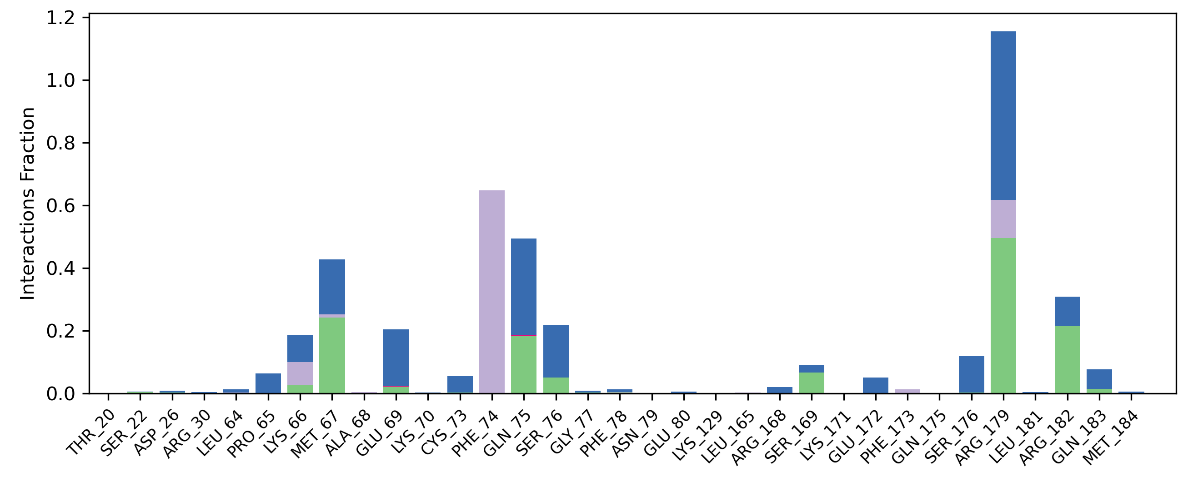


Supplementary Figure 4: Graphs showing molecular complex stability of GarA with IL-6: A. RMSF and B. Interaction residues

- 1. **MCP-1-GarA interaction**


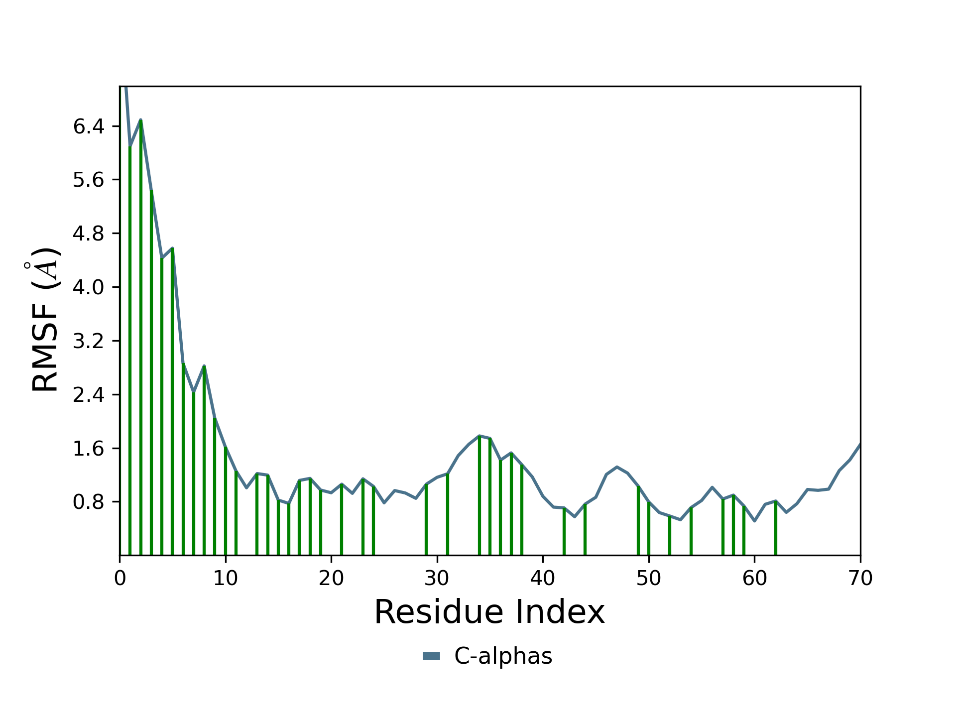


**A**

**B**


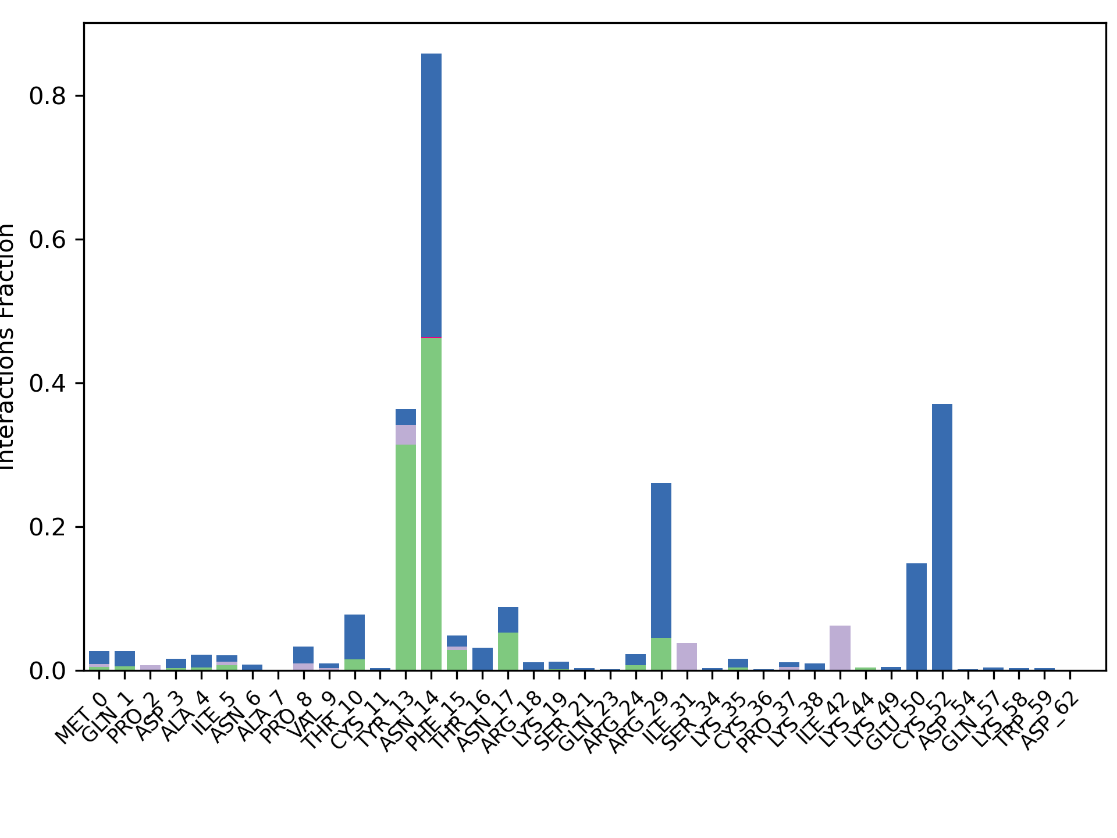


Interactions Fraction

Supplementary Figure 5: Graphs showing molecular complex stability of GarA with MCP-1: A. RMSF and B. Interaction residues
